# SDC1-TGM2-FLOT1-BHMT complex determines radiosensitivity of glioblastoma by influencing the fusion of autophagosomes with lysosomes

**DOI:** 10.7150/thno.81999

**Published:** 2023-06-26

**Authors:** Liang Zeng, Wang Zheng, Xinglong Liu, Yuchuan Zhou, Xiaoya Jin, Yuqi Xiao, Yang Bai, Yan Pan, Jianghong Zhang, Chunlin Shao

**Affiliations:** 1 Institute of Radiation Medicine, Shanghai Medical College, Fudan University, Shanghai 200032, China.; 2 Department of Radiation Oncology, The First Affiliated Hospital of Nanjing Medical University, Nanjing 210009, China.

**Keywords:** SDC1, TGM2, glioblastoma, radioresistance, autophagy

## Abstract

**Rationale:** Glioblastoma (GBM) is the most common and malignant primary brain tumor in adults. Radiotherapy has long been an important treatment for GBM. Despite recent advances in tumor radiotherapy, the prognosis of GBM remains poor due to radioresistance. Autophagy has been reported as a basic factor to prolong the survival of tumor under radiation stress, but the molecular mechanism of how autophagy contributes to GBM radioresistance was still lacking.

**Methods:** We established radioresistant GBM cells and identified their protein profiles by Tandem mass tag (TMT) quantitative proteomic analysis, then chose the radioresistant genes based on the TMT analysis of GBM cells and differentially expressed genes (DEGs) analysis of GEO database. Colony formation, flow cytometry, qPCR, western blotting, mRFP-GFP-LC3, transmission electron microscopy, immunofluorescence, and co-IP assays were conducted to investigate the regulation mechanisms among these new-found molecules.

**Results:** Syndecan 1 (SDC1) and Transglutaminase 2 (TGM2) were both overexpressed in the radioresistant GBM cells and tissues, contributing to the dismal prognosis of radiotherapy. Mechanically, after irradiation, SDC1 carried TGM2 from cell membrane into cytoplasm, and transported to lysosomes by binding to flotillin 1 (FLOT1), then TGM2 recognized the betaine homocysteine methyltransferase (BHMT) on autophagosomes to coordinate the encounter between autophagosomes and lysosomes.

**Conclusions:** The SDC1-TGM2-FLOT1-BHMT copolymer, a novel member of the protein complexes involved in the fusion of lysosomes and autophagosomes, maintained the autophagic flux in the irradiated tumor cells and ultimately enhanced radioresistance of GBM, which provides new insights of the molecular mechanism and therapeutic targets of radioresistant GBM.

## Introduction

Glioblastoma is the most common and lethal primary malignancy of the central nervous system (CNS) with high recurrence and mortality rates, severely threatening human life worldwide. Current standard treatment strategies include complete or sub-complete surgical resection, alkylating agent administration chemotherapy, and radiation therapy 1. Alternative treatment such as immunotherapy and targeted therapy are also applied 2. Despite recent advances in the management of GBM, no treatment is curative for GBM and nearly all patients recur inevitably with a median survival less than 15 months and 5-year survival rate less than 10% 3. Among different treatments, radiotherapy is highly recommended for up to 50% patients as postoperative radiotherapy or neoadjuvant radiotherapy 4. Accumulating evidence has revealed that the weak response of glioma cells to irradiation leads to poor therapeutic outcomes. Hence, the development of effective strategies to sensitize glioma cells to irradiation with fewer adverse effects is urgently anticipated.

Autophagy is an adaptive process by which aggregated proteins or damaged organelles are sequestered by double-membrane autophagosomes and degraded in autolysosomes, allowing cells to cope with stress and preserve cellular homeostasis under physiological conditions. Previous study has found that autophagy is highly activated in glioblastoma 5. Irrespective of molecular sub-classification, nearly all GBM are treated with radiation and temozolomide (TMZ), which are known to induce autophagy that contributes to tumor cell survival 6. Thus, the suppression of autophagic activity may sensitize GBM to radiotherapy.

Syndecan 1 (SDC1, also named CD138) is one of the type I single transmembrane proteoglycans and works in maintaining normal cell morphology, interacting with extracellular and intracellular protein pools as well as mediating signaling transduction upon environmental stimuli 7. The critical role of SDC1 in promoting tumorigenesis and metastasis has been increasingly recognized in various cancer types 8-10, implying a promising potential of utilizing SDC1 as a novel target for cancer therapy. Transglutaminase 2 (TGM2) is a stress-responsive gene that regulates multiple cellular processes during normal cell development. It was reported that TGM2 could promote autophagy and was upregulated in astrocytes of ex vivo and in vivo models of hepatic encephalopathy (HE), as well as in post mortem brain samples of liver cirrhosis patients with HE 11. Flotillin 1 (FLOT1), an essential marker of lipid rafts, is involved in various procedures, including cell proliferation, migration, cell adhesion, survival, differentiation, endocytosis, signal transduction and membrane trafficking 12. Currently, growing evidence demonstrates that lipid rafts could promote initiation of autophagy 13, 14. Betaine homocysteine methyltransferase (BHMT), one of many cytosolic proteins found in the mammalian autophagosome, has recently been developed as an end-point marker of mammalian autophagy 15.

In this study, we aimed to understand the relationship among SDC1, TGM2, FLOT1 and BHMT in modulating glioma autophagy and investigated their roles in regulating the radiosensitivity of glioblastoma by affecting the fusion of autophagosomes and lysosomes. The results could inform a novel potential approach for glioma radiotherapy.

## Materials and Methods

### Cell culture and irradiation

Human glioblastoma cell lines of U251 and U87 was purchased from Cell Bank of Chinese Academy of Science (Shanghai, China). In our previous study, U251 cell line received 2 Gy X-ray/day for 30 fractions (5 fractions/weekly in general) with a total dose of 60 Gy to construct a stable radioresistant cell line U251R 16. U251 and U251R were cultured in DMEM medium (Gibco, Thermo Fisher Scientific, Waltham, MA, USA) supplemented with 10% fetal bovine serum (Gibco Invitrogen, Grand Island, NY, United States), 100 units/mL penicillin and 100 mg/mL streptomycin (Gibco). All cells were incubated at 37 °C in 5% CO_2_ and subcultured every 2 to 3 days. Cells in log-phase were irradiated with a dose rate of 0.883 Gy/min X-ray (X-RAD 320, PXI Inc., North Branford, CT, USA; 12 mA, 2 mm aluminum filtration) at room temperature. Testing for mycoplasma was performed once a month.

### Colony formation assay

The radiosensitivity of different cell lines was assessed by cell colony formation assay. Briefly, cells were trypsinized to generate single-cell suspension and planted in six-well plates at a proper density. After full attachment, they were exposed to 0, 2, 4, 6, and 8 Gy of X-rays. Approximately 2 weeks after radiation, cell colonies were fixed with methanol for 20 min and stained with 0.1% crystal violet for 30 min to count the colonies with more than 50 cells. The cell survival curve was fitted with a single hit multitarget model using GraphPad Prism 8 (GraphPad software, San Diego, CA, USA). Sensitization enhancement ratio (SER) was expressed as an enhancement ratio determined at a survival fraction (SF) of 37%.

### Flow cytometry detection of apoptosis

After 24 h of irradiation with 4 Gy X-rays, the cells were washed with cold PBS and collected in 50 µL of the suspension buffer containing 2.5 µL Annexin V-FITC and 2.5 µL PI (TransGen Biotech, Beijing, China), then 200 µL buffer was added to the solution. After incubation for about 15 min in the dark at room temperature, cell apoptosis was detected using flow cytometry (Beckman CytoFLEX, CA, USA) and analyzed with FlowJo software (version 10). Viable cells display negative FITC Annexin-V and PI (FITC^-^/PI^-^), cells in early apoptosis have positive FITC Annexin-V and negative PI (FITC^+^/PI^-^), cells in late apoptosis have positive FITC Annexin-V and PI (FITC^+^/PI^+^), and dead cells have negative FITC Annexin-V and positive PI (FITC^-^/PI^+^).

### RNA extraction and RT-qPCR assay

Total cellular RNA was extracted using TRIzol reagent (Invitrogen, San Diego, CA, USA). Reverse transcription of total RNA to cDNA was performed using a FastKing RT Kit (with gDNase) (Tiangen Biotechnology, Beijing, China). RT-qPCR was carried out in 20 μl reaction reagents using SuperReal PreMix Plus (SYBR Green) (Tiangen Biotechnology, Beijing, China) with the MX3000P platform according to the manufacturer's protocol. The primers used in the RT-qPCR assays are listed in Supplementary Table S1.

### Western blotting assay

Total cellular proteins were extracted using RIPA buffer with protease inhibitor (Beyotime Biotechnology, Shanghai, China). Then an equal amount of protein was separated by sodium dodecyl sulfate polyacrylamide gel electrophoresis (SDS-PAGE) on a 10% gel and transferred to a polyvinylidene difluoride (PVDF) membrane (Millipore, Bedford, MA, USA). The membrane was blocked with 5% non-fat milk in Tris buffer saline/Tween 0.05% (TBST) for 2 h and incubated overnight at 4 °C with appropriate primary antibodies. After incubation with HRP-conjugated secondary antibodies, protein bands were detected by the enhanced chemiluminescence system (ECL kit, Millipore, St. Louis, MO, United States), and band images were analyzed with a ChemiDoc XRS system (Bio-Rad Laboratories Inc., Hercules, CA, USA). Antibodies are listed in Supplementary Table S2.

### Transient transfection

To overexpress SDC1 and TGM2 (oeSDC1 and oeTGM2), the cells were transfected with pcDNA3.1 (Invitrogen™, USA) or its negative control (oe-NC). Meanwhile, two specific siRNAs of SDCA, TGM2, FLOT1 or BHMT were designed and offered by GenePharma (Shanghai, China). U251R and U87 cells were transferred with SDC1 siRNA (siSDC1), TGM2 siRNA (siTGM2), FLOT1 siRNA (siFLOT1), BHMT siRNA (siBHMT) or negative control siRNA (siNC) using riboFECT CP Transfection Agent (RiboBio, Guangzhou, China), according to the manufacture's protocol. At 24 h after transfection, cells were exposed to irradiation or other treatments. The interfering efficiency was identified by Western blot assay at 24-72 h after transfection. The RNA interference sequences were listed in Supplementary Table S3.

### Tandem Mass Tag (TMT) quantitative proteomic analysis

Total proteins were extracted from U251 and U251R cells and their concentrations were detected with the bicinchoninic acid assay (BCA Protein Assay Kit; Beyotime Biotechnology, Haimen, China). A quantity of 0.2 mg of protein from each sample was used for TMT analysis following the manufacturer's protocol (Genechem, Shanghai, China). The quantitative proteomics analysis was performed with a high-resolution mass spectrometer Q Exactive plus (Thermo Fisher Scientific) and the database Uniprot_HomoSapiens_20386_20180905 (website: http://www.uniprot.org accessed on 19 March 2019) was used for protein sequence analysis.

### Bioinformatics analysis

Three datasets were used for the bioinformatics analysis. In addition to the TMT data obtained from our own cell samples, two datasets obtained from the Gene Expression Omnibus (GEO) data sets (https://www.ncbi.nlm.nih.gov/geo/, accessed 20 April 2022) (GSE76070, GSE10547) were also used to analyze the differentially expressed genes (DEGs) between irradiated and non-irradiated groups and between GBM tumoral and normal tissues. The DEGs analysis was performed using the Bioconductor package limma and the results were showed by heatmap using R programming language.

### Autophagy flux assay

Autophagy was examined by analyzing the formation of fluorescent puncta of autophagosomes in the cells transfected with mRFP-GFP-LC3-tagged adenovirus (Hanbio Biotechnology Co., Shanghai, China). Briefly, cells were plated at a density of 2 × 10^5^ per well in 24-well plate and cultured overnight. After 2 h of transfection with the adenovirus (diluted in serum-free medium), the cells were cultured in fresh medium for 48 h then washed with pre-cooled PBS twice, fixed and stained with DAPI. The intracellular autophagy was observed with the high-content imaging system. When the fusion of autophagy and lysosome occurs, LC3-GFP fluorescence is quenched and only red fluorescence can be detected. After merging the red and green fluorescence images, yellow spots in the cell images symbolize the puncta of autophagosomes. For quantitative analyses, the numbers of yellow (autophagosomes) and red only puncta (autolysosomes) and ratio of autolysosomes to autophagosomes were quantified per GBM cell from random fields in three independent experiments (totally n = 50 cells).

### Transmission electron microscopy observation

Transmission electron microscopy (TEM) was carried out for the ultrastructural analysis of autophagosome/autophagolysosome. The GBM cells with different pretreatments were fixed in ice-cold 2.5% glutaraldehyde at 4 °C overnight and then fixed in 1% osmium tetroxide (OsO4) at 4 °C for 2 h. The samples were subsequently rinsed with water and dehydrated in a graded series of alcohol (50, 75, and 100%), and then embedded in Epon812 epoxyresin. The embedded samples were sectioned, stained with a uranyl acetate solution, and then with an alkaline lead citrate solution followed by examination under a TEM (JEM‐1200EX, JEOL, Ltd., Japan).

### Autophagy induction and inhibition assays

Rapamycin (50 nM) was used to activate autophagy, and 3-MA (100 μM) or bafilomycin A1 (100 nM) was used to inhibit autophagy. The GBM cells were pre-treated with rapamycin, 3-MA or bafilomycin A1 for 2 h and then irradiated with X-rays. All reagents were dissolved and stored in DMSO.

### Immunofluorescence assay

Cells in 70-80% confluence were irradiated with 4 Gy X-rays. Two or four hours after irradiation, cells were washed with cold PBS, fixed with 4% paraformaldehyde for 10 min, permeabilized with 0.5% Triton X-100 for 10-15 min and then incubated in 0.1% PBS-Tween solution containing 1% BSA, 10% normal goat serum, and 0.3 M glycine for 1 h at room temperature to block non-specific protein-protein interaction. Cells were then incubated with primary antibodies detecting SDC1, TGM2, FLOT1 and BHMT (1:200, Cell Signaling Technology) overnight at 4 °C, and followed by Alexa Fluor 488, 555 or 647-conjugated secondary antibody (1:1000, Cell Signaling Technology) for 1 h in dark. After being well washed with PBS, the cells were mounted with 1.43 μM DAPI (Beyotime Biotechnology) to stain the cell nuclei. Finally, the cell fluorescence images were photographed with a high content screening system (ImageXpress Micro 4, Molecular Devices, San Jose, CA, USA). For quantitative analyses, the fluorescence spot numbers or relative average optical density of the expressions of SDC1, TGM2, FLOT1, BHMT, LC3, LAMP2 proteins and their merged regions in each GBM cell were counted with the ImageJ software (National Institutes of Health, USA). Colocalization was defined if fluorescent signals of two or three puncta overlapped by >70%. A total of 50 cells from random fields in three independent experiments were counted for each analysis.

### Co-immunoprecipitation (Co‑IP) assay

Whole cell lysates were collected according to the manufacturer's instruction (Beyotime Biotechnology) and then centrifuged at 10,000 g for 10 min at 4°C. Then 1 mL supernatant was incubated with 1 μg anti-SDC1, anti-FLOT1, anti-Flag antibody (1:100, Proteintech Group), or anti-IgG antibody (1:100, Cell signaling Technology) for 16 h followed by addition of 20 μL protein A/G plus agarose beads (Santa Cruz Biotechnology) and incubated overnight at 4 °C. Protein samples were spun down, washed four times with immunoprecipitation buffer and heated for 10 min at 100 °C prior to loading on an SDS-PAGE gel for Western blot assay. Finally, the protein bands were visualized by ChemiDoc XRS system (Bio-Rad Laboratories Inc.). Antibodies are listed in supplementary Table S2.

### Plasmid construction

We constructed a plasmid encoding Flag-tagged wild-type SDC1 and a plasmid with domain-mutant fragment (the amino acids in the first constant range and variable region of cytoplasmic domain, also known as SDC1 amino acids 277-306, mutated to alanine) and site-directed mutants in the second constant range (glutamine 307, phenylalanine 308 and tyrosine 309 mutated to alanine, alanine 310 deleted) of SDC1 in the pcDNA3.1(-) backbone (Genechem). Each plasmid was transiently transfected into U251R and U87 cells by Lipofectamine 3000 (Invitrogen, L3000008). After 24 h of transfection, cells were irradiated, and their proteins were prepared for immunoprecipitation and immunoblot assays.

### Statistical analysis

All experiments were repeated at least three times and the data are presented as the mean ± SD. The difference between indicated groups was evaluated by Student's t-test or one-way analysis of variance (ANOVA) using GraphPad Prism 8 (GraphPad software, San Diego, CA, USA). P < 0.05 was considered statistically significant.

## Results

### High levels of SDC1 and TGM2 closely associated with the radioresistance and poor prognosis of GBM

**Figure 1:**
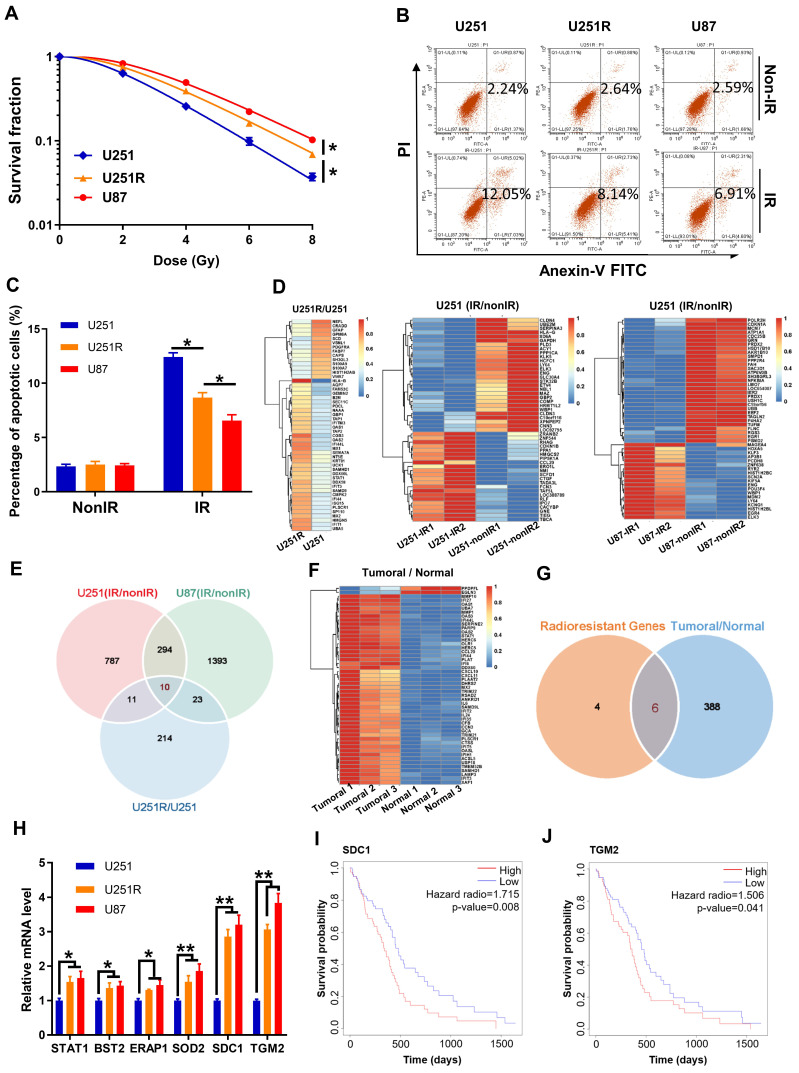
** High levels of SDC1 and TGM2 closely associated with the radioresistance and poor prognosis of GBM.** (**A**) Clonogenic survival fractions of U87, U251R and U251 cells exposed to 0, 2, 4, 6, and 8 Gy of X-rays**. (B)** Flow cytometry assay of apoptosis in U251, U251R and U87 cells with or without 4 Gy irradiation.** (C)** Quantitative data of apoptosis induction. (**D**) Heatmaps of the top 50 differentially expressed proteins between U251 and U251R cells (left, fold change ≥ 1.3, *P* < 0.05), and top 50 differentially expressed genes (DEGs) of the cells (U251 and U87) before and after irradiation (middle and right, fold change ≥ 1.5, *P* < 0.05). (**E**) Wayne chart of the overexpressed genes in irradiated U251 (pink), irradiated U87 (green) and radioresistant U251R (blue) cells. The intersection contains 10 genes. (**F**) Heatmap of the top 50 DEGs between tumoral and normal brain tissue from patients with GBM (fold change ≥ 1.5, *P* < 0.05). (**G**) Wayne chart of the radioresistant genes (yellow, intersection part of the above Wayne chart) and the overexpressed genes in the tumoral tissue (blue). The intersection contains 6 genes. (**H**) Expression quantification by qRT-PCR of the 6 genes selected from Wayne chart in U251, U251R and U87 cells. (**I-J**) Kaplan-Meier analysis of the relationship between SDC1 (**I**) / TGM2 (**J**) expression and overall survival (OS) of GBM patients (data from ExSurv). * *P* < 0.05, ** *P* < 0.01.

We have successfully established a radioresistant cell line (named as U251R) by long-term fractional X-ray irradiation with a total dose of 60 Gy on U251 cells 16, and verified that the radioresistance of U251R was significantly higher than its parental U251 cells, while still lower than U87 cells (Figure 1A-C). The proteins from U251 cells and its radioresistant counterpart U251R were collected and subjected to TMT quantitative proteomic analysis to determine the differentially expressed genes (DEGs) between radioresistant cells and their parents. Total 437 DEGs were identified, among which 258 genes were upregulated, and 179 genes were downregulated (fold change ≥ 1.3, *P* < 0.05). To further explore radioresistant genes, we also analyzed the DEGs of the glioma cells before and after irradiation using GEO dataset GSE10547. Compared to non-irradiated cells, 717 genes were downregulated, and 1102 genes were upregulated in irradiated U251 cells, and 1336 genes were downregulated and 1720 genes were upregulated in irradiated U87 cells (fold change ≥ 1.5,* P* < 0.05). The top 50 DEGs mentioned above were illustrated by heatmaps, respectively (Figure 1D). Venn diagram (Figure 1E) revealed that 10 genes were collectively upregulated in the above three groups (U251R versus U251, irradiated U251 versus non-irradiated U251, and irradiated U87 versus non-irradiated U87). In addition, we also analyzed the DEGs between glioma tissues and adjacent normal tissues using GEO dataset GSE76070. Compared to normal brain tissues, there were 394 highly expressed genes while 204 lowly expressed genes in tumoral tissues (fold change ≥ 1.5, *P* < 0.05). The top 50 DEGs were illustrated by a heatmap (Figure 1F). When we intersected the high-expression genes in the clinical tumor tissues with the aforementioned 10 co-upregulated genes (Figure 1E), 6 genes were obtained, which might be related to the radioresistance of glioma (Figure 1G, S1A). Among these 6 genes, PCR assay identified that both SDC1 and TGM2 had outstanding expression levels in the radioresistant glioma cells (Figure 1H). Furthermore, according to the Kaplan-Meier analysis of overall survival (OS), the prognosis of GBM patients with high expression level of SDC1 and TGM2 were markedly worse (Figure 1I-J). These bioinformatics analyses imply that the high level of SDC1 and TGM2 is a key factor of tumor radioresistance and may affect the outcome of radiotherapy of glioma patients.

### Inhibition of SDC1 or TGM2 enhanced radiosensitivity of GBM cells

**Figure 2:**
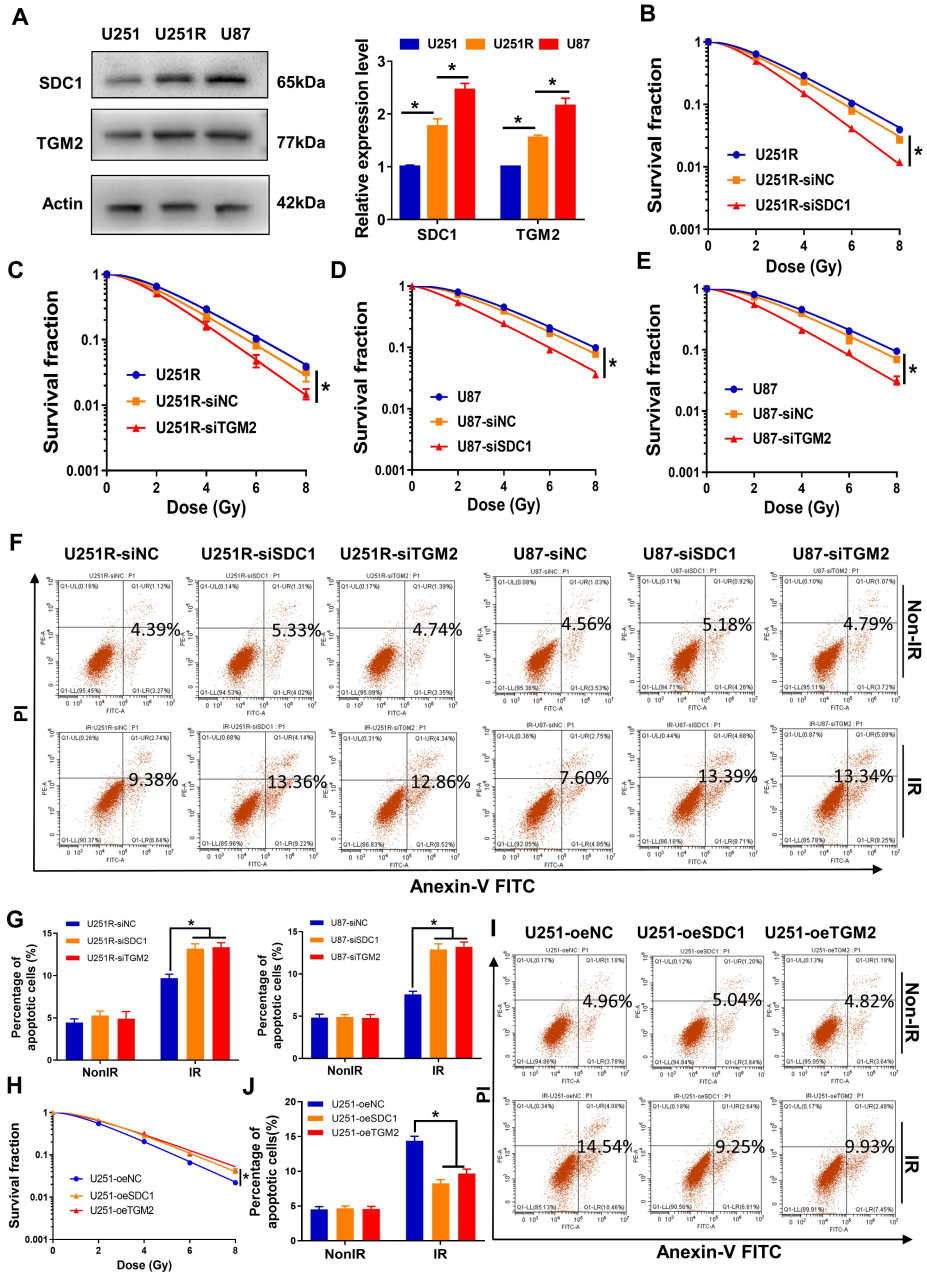
** Effect of SDC1 and TGM2 on radiation sensitivity.** (**A**) Western blot analysis (left) and quantitation (right) of SDC1 and TGM2 expression levels in U251, U251R and U87 cells. Actin was used as a loading control. (**B-E**) Clonogenic survivals of U251R (**B-C**) and U87 (**D-E**) cells transfected with siSDC1 (**B**, **D**) or siTGM2 (**C**, **E**) after irradiation. (**F-G**) Flow cytometry assay of apoptosis (**F**) and quantitation (**G**) in different siRNAs transfected U251R and U87 cells with or without 4 Gy irradiation. (**H**) Clonogenic survivals of U251-oeNC, U251-oeSDC1 and U251-oeTGM2 cells after irradiation. (**I-J**) Flow cytometry assay of apoptosis (**I**) and quantitation (**J**) in U251-oeNC, U251-oeSDC1 and U251-oeTGM2 cells with or without 4 Gy irradiation. * *P* < 0.05, ** *P* < 0.01.

To further disclose the relationship of SDC1 and TGM2 with radiosensitivity of GBM, we detected the cellular expression levels of SDC1 and TGM2 by Western blot assay and found that they increased orderly in U251, U251R and U87 cells (Figure 2A), which has a positive correlation with the radiosensitivity as shown in Figure 1A. Here, we applied U87 cells as naturally radioresistant cells and U251R as acquired radioresistant cells, both compared to U251. When the radioresistant cells U251R and U87 were transfected by SDC1 siRNA (siSDC1) or TGM2 siRNA (siTGM2), and the radiosensitive cells U251 were overexpressed with SDC1 (oeSDC1) or TGM2 (oeTGM2), with effective interfering efficiencies verified by Western blot assay (Figure S1B-E), the cell responses to radiation were changed remarkably i.e., U251R and U87 cells were sensitized by siSDC1 and siTGM2 (Figure 2B-G), and U251 acquired more radioresistance by oeSDC1 and oeTGM2 (Figure 2H-J). These results indicated that the high levels of SDC1 and TGM2 contributed to the radioresistance of glioma cells.

### Autophagy regulates the radiosensitivity of GBM cells

**Figure 3:**
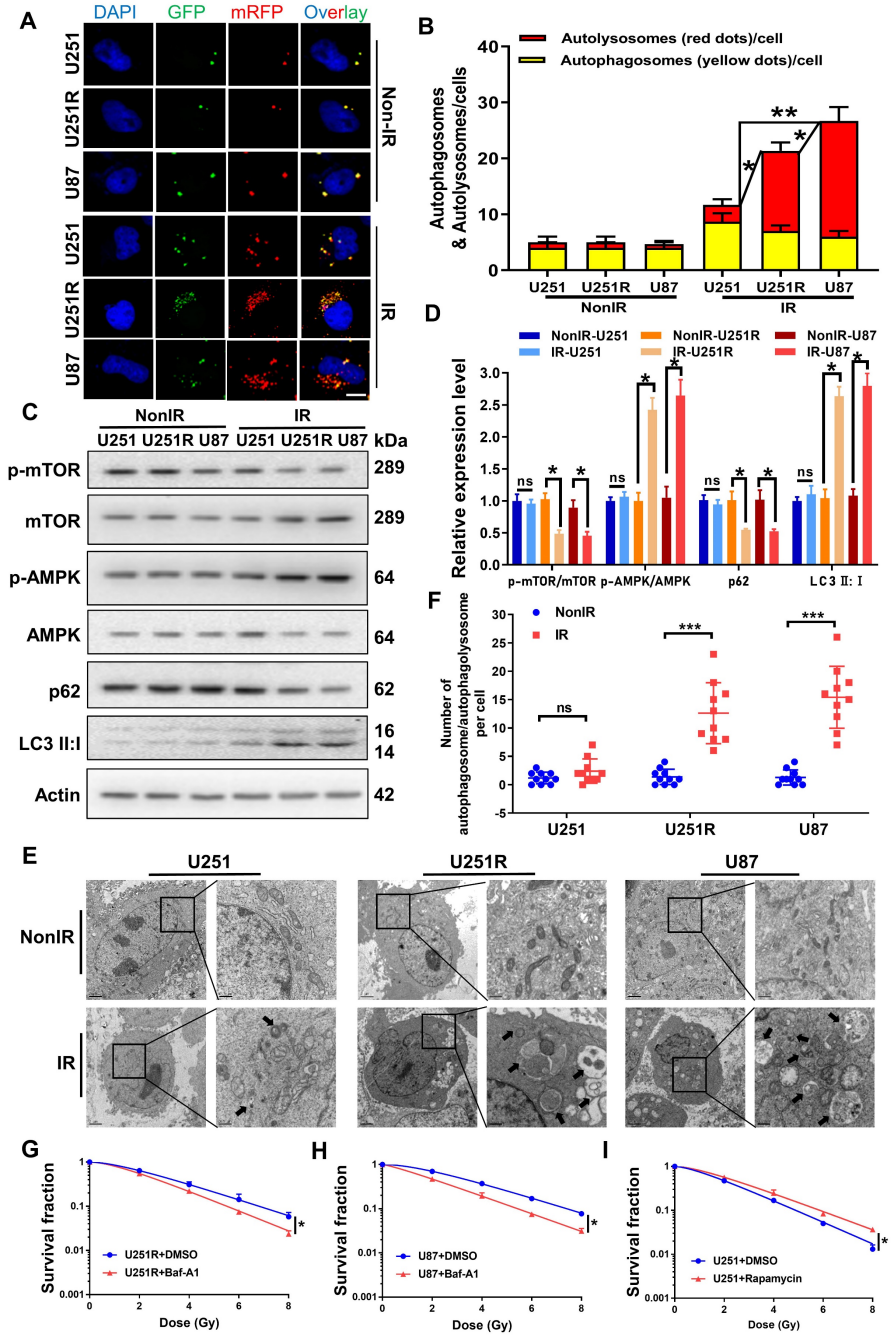
** Autophagic activity was positively correlated with GBM radioresistance.** (**A-B**) Fluorescence images (**A**) of U251, U251R and U87 cells transfected mRFP-GFP-LC3-tagged adenovirus (×40) after 4 Gy irradiation. Red dots indicate autolysosomes while yellow dots indicate autophagosomes in overlays. Nuclei were stained with DAPI. Scale bars: 10 μm. The average number of autophagosomes and autolysosomes in each indicated cell was quantified (**B**). A total of 50 cells from random fields of each group were counted for each analysis. (**C-D**) Western blot analysis (**C**) and quantitation (**D**) of p-mTOR/mTOR, p-AMPK/AMPK, p62 and LC3-Ⅱ:Ⅰ protein levels in U251, U251R and U87 cells at 4 h after 4 Gy irradiation. (**E**) TEM examination showing the autophagosome/autophagolysosome in U251, U251R and U87 cells with or without 4 Gy irradiation. Autophagosome/autophagolysosome were indicated by black arrows. Scale bar: 2 μm (left) and 0.5 μm (right) in each group. (**F**) Average numbers of autophagosome /autophagolysosome-like vesicles in U251, U251R and U87 cells. Ten cells in random fields were counted for each group. (**G-H**) Clonogenic survivals of U251R and U87 cells that were pre-treated with 100 nM bafilomycin A1 (Baf-A1) for 2 h and then irradiated with X-rays. (**I**) Survival fractions of U251 cells that pre-treated with 50 nM rapamycin for 2 h and then irradiated with X-rays. * *P* < 0.05, ** *P* < 0.01 and ns *P* > 0.05.

Our previous study provided evidence that autophagic activity was positively correlated with the radioresistance of tumor cells 16, 17. Therefore, we transfected GBM cells with mRFP-GFP-LC3-tagged adenovirus to label autophagosomes and to determine whether autophagy is involved in the regulation of GBM radioresistance. The results showed that all the three GBM cell lines contained a very few autolysosomes (red dots in overlays) before irradiation (Figure 3A-B). After irradiation, the number of autolysosomes significantly increased in U251R and U87 cells, while it remained nearly unchanged in U251 cells, indicating that the irradiation enhanced autophagic flux in U251R and U87 cells, but not U251 cells. Western blot assay and transmission electron microscopy (TEM) examination had consistent results. We detected the phosphorylation levels of monophosphate-activated protein kinase (AMPK), mammalian target of rapamycin (mTOR) (trigger of autophagy), the relative expression levels of LC3 and p62 (maturation-promoter of autophagosome), and found that the ratio of p-AMPK/AMPK and LC3II/LC3I increased while p-mTOR/mTOR and p62 decreased, illustrating enhanced autophagic flux in U251R and U87 cells after irradiation (Figure 3C-D). Moreover, the TEM images also revealed that the amount of autophagosome/autophagolysosome was increased in the radioresistant cells compared to the radiosensitive cells after irradiation (Figure 3E-F). Based on these results, we speculated that the radioresistance of U251R and U87 cells may depend on the increased levels of autophagy that protected the cells from radiation-induced cell death. To further verify this hypothesis, we treated two radioresistant GBM cell lines (U251R and U87) with bafilomycin A1 (Baf-A1, autophagy inhibitor) and found that it decreased radioresistance (Figure 3G-H). In contrast, the addition of Rapamycin (autophagy promoter) to radiosensitive GBM cells (U251) increased its radioresistance (Figure 3I). Therefore, autophagy could regulate the radiosensitivity of GBM cells.

### SDC1 and TGM2 mediated radioresistance of GBM through autophagy

Above results reveal that SDC1, TGM2 and autophagy can regulate the radiosensitivity of GBM cells. An interesting question is whether SDC1 and TGM2 have any correlation with autophagy in regulating GBM radiosensitivity? To determine this, we first transfected siSDC1 and siTGM2 into U251R and U87 cells, then detected the autophagic flux using western blot assay, mRFP-GFP-LC3-tagged adenovirus, and TEM examination.

**Figure 4:**
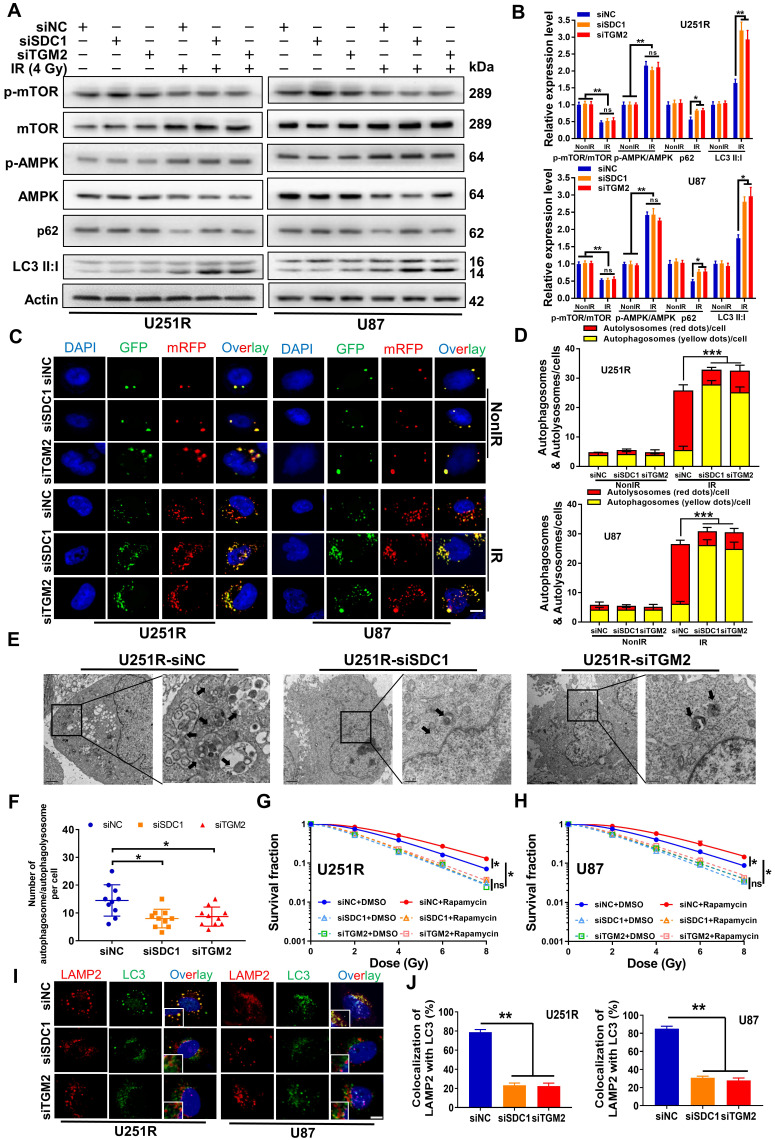
** SDC1 and TGM2 mediated radioresistance of GBM through autophagy.** (**A-B**) Western blot analysis (**A**) and quantitation (**B**) of p-mTOR/mTOR, p-AMPK/AMPK, p62 and LC3-II:I protein levels in different siRNAs transfected U251R and U87 cells at 4 h after 4 Gy irradiation. (**C-D**) Fluorescence images of U251R and U87 cells transfected with different siRNAs and mRFP-GFP-LC3-tagged adenovirus (×40) after 4 Gy irradiation. Red dots indicate autolysosomes while yellow dots indicate autophagosomes in overlays. Nuclei were stained with DAPI. Scale bars: 10 μm. The average number of autophagosomes and autolysosomes in each indicated cell was quantified. A total of 50 cells from random fields of each group were counted for each analysis. (**E**) TEM examination showing the autophagosome/autophagolysosome in irradiated U251R cells with control or knockdown of SDC1/TGM2. Autophagosome/autophagolysosome were indicated by black arrows. Scale bar: 2 μm (left) and 0.5 μm (right) in each group. (**F**) Average numbers of autophagosome/autophagolysosome-like vesicles in irradiated U251R cells transfected with different siRNAs. Ten cells in random fields were counted for each group. (**G-H**) Clonogenic survivals of U251R (**G**) and U87 (**H**) cells transfected with siNC, siSDC1 or siTGM2. Cells were pre-treated with 50 nM rapamycin for 2 h and then irradiated with X-rays. (**I-J**) Fluorescence images of LAMP2 (red) and LC3 (green) in different siRNAs transfected U251R and U87 cells at 4 h after 4 Gy irradiation. Nuclei were stained with DAPI (blue). Colocalization of LC3 and LAMP2 was quantified. A total of 50 cells from random fields were counted for each analysis. Scale bars: 10 μm. * *P* < 0.05, ** P < 0.01, *** *P* < 0.001, and ns *P* > 0.05.

The results showed that the autophagy levels of U251R and U87 cells under irradiation were reduced by knocking-down SDC1 and TGM2 (Figure 4A-F), and the radioresistance was decreased accordingly (Figure 2B-E). Meanwhile, the overexpression of SDC1 and TGM2 (Figure S1D-E) increased autophagy (Figure S2A-D) and radioresistance (Figure 2H-J) in U251 cells. But the enhanced radioresistance was abolished by autophagy inhibitors, 3-MA and Baf-A1 (Figure S2E-G). To sum up, these results fully demonstrated that SDC1 and TGM2 enhanced the radioresistance of GBM cells by increasing the level of autophagy.

Notably, we observed no restoration of radioresistance in GBM cells with knocked-down SDC1 and TGM2 after treatment with the autophagy promoter Rapamycin (Figure 4G-H). To better explain this interesting phenomenon, we further examined the autophagic flux in these cells. Western blot analysis (Figure S3A-B) showed that in U251R and U87 cells, the ratio of p-AMPK/AMPK decreased and the ratio of p-mTOR/mTOR increased following Rapamycin treatment, regardless of whether SDC1 or TGM2 were knocked down. These two indicators of autophagy initiation stage suggested that the promotion of Rapamycin on the initiation of autophagy in GBM cells was independent of SDC1 and TGM2. However, the situation of other two markers, p62 and LC3II/LC3I (indicators of autophagy maturation stage), were different. The expression level of p62 decreased in the cells transfected with siNC after Rapamycin treatment, but it increased in the cells transfected with siSDC1 and siTGM2. It is generally believed that the expression level of p62 is negatively correlated with the level of autophagy. As for LC3II/LC3I, Rapamycin increased this ratio regardless of whether SDC1 or TGM2 was knocked down, but the increase was more significant in the siSDC1 and siTGM2 groups. Generally, when the autophagy level was upregulated or the autolysosome was blocked, the ratio of LC3II/LC3I was increased. Combining the change of p62, we hypothesized that the increase of LC3II/LC3I in siNC-transfected cells was due to an overall increase of autophagy flow, while in siSDC1- or siTGM2-transfected cells, the increase of LC3II/LC3I was due to the block of autolysosome related process. This conjecture was further confirmed by the subsequent mRFP-GFP-LC3 assay (Figure S3C-D), which showed that following addition of Rapamycin, the number of autolysosomes (red dots in overlays) increased in siNC-transfected cells, while in siSDC1- or siTGM2-transfected GBM cells, only the number of autophagosomes (yellow dots in overlays) increased and the number of autolysosomes (red dots in overlays) remained at a low level, indicating that knockdown of SDC1 and TGM2 inhibited the formation of autolysosomes. To illustrate this more intuitively, we compared the colocalization of endogenous LC3 with lysosomal associated membrane protein 2 (LAMP2, a lysosomal marker protein) in siNC-, siSDC1- or siTGM2-transfected cells by immunofluorescence assay. Colocalization was defined if fluorescent signals of two puncta overlapped by >70%. We found that in both U251R and U87 cells, either SDC1- or TGM2-knockdown could significantly reduce the radiation-induced clusters of lysosomal LAMP2 and autophagosomal LC3, indicating a block of the fusion of autophagosomes with lysosomes (Figure 4I-J). Now, back to the initial question, we know that the deficiency of SDC1 and TGM2 could inhibit the formation of autolysosome in the maturation stage of autophagy flow, while Rapamycin promotes the formation of autophagosomes in the initiation stage of autophagy flow. That is why the use of Rapamycin did not restore the radioresistance of GBM cells transfected with siSDC1 and siTGM2. Taken together, the findings indicated that SDC1 and TGM2 could upregulate the radioresistance of GBM cells by promoting the fusion of autophagosomes with lysosomes to enhance the level of autophagy after irradiation.

### FLOT1 and BHMT mediated radioresistance of GBM through autophagy

In exploring molecular interactions during the fusion of autophagosomes and lysosomes, it is known that FLOT1 is a lipid raft-associated scaffolding protein, physiologically acting as an endosomal transporter of membrane proteins, which could localize on endolysosomes 18, 19. Intersecting with the endosomal-lysosomal system, the autophagy pathway transports organelles to lysosome for degradation. Besides, BHMT was considered as a potential cargo-based, end-point marker for mammalian autophagy 15. Survival analysis also suggested the clinical value of FLOT1 and BHMT.

**Figure 5:**
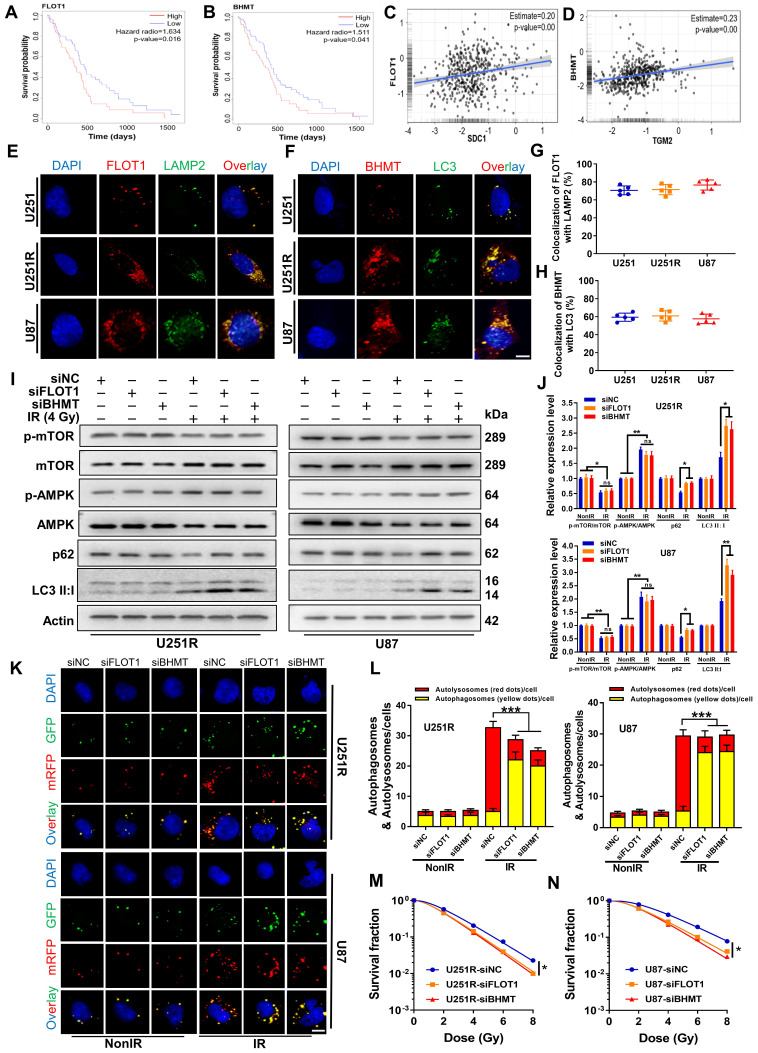
** Inhibition of FLOT1 or BHMT enhanced radiosensitivity of GBM cells by reducing autophagy level.** (**A-B**) Kaplan-Meier analysis of the relationship between FLOT1 (**A**) /BHMT (**B**) expression and overall survival (OS) of GBM patients (data from ExSurv). (**C-D**) The correlations between FLOT1 and SDC1 (**C**), BHMT and TGM2 (**D**) were analyzed using GlioVis database. (**E-H**) Immunostaining images (left) of FLOT1 (red) and LAMP (green) in U251R and U87 cells at 4 h after 4 Gy irradiation. Nuclei were stained with DAPI (blue) (**E**). Colocalization of FLOT1 and LAMP2 was quantified (**G**). Immunostaining images (up) of BHMT (red) and LC3 (green) in U251R and U87 cells at 4 h after 4 Gy irradiation. Nuclei were stained with DAPI (blue) (**F**). Colocalization of BHMT and LC3 was quantified (**H**). A total of 50 cells from random fields of each group were counted for each analysis. (**I-J**) Western blot analysis (**I**) and quantitation (**J**) of p-mTOR/mTOR, p-AMPK/AMPK, p62 and LC3-Ⅱ:Ⅰ protein levels in different siRNAs transfected U251R and U87 cells at 4 h after 4 Gy irradiation. (**K-L**) Fluorescence images of U251R and U87 cells transfected with different siRNAs and mRFP-GFP-LC3-tagged adenovirus (×40) after 4 Gy irradiation. Red dots indicate autolysosomes while yellow dots indicate autophagosomes in overlays. Nuclei were stained with DAPI. Scale bars: 10 μm. The average number of autophagosomes and autolysosomes in each indicated cell was quantified. A total of 50 cells from random fields of each group were counted for each analysis. (**M-N**) Clonogenic survivals of U251R (**E**) and U87 (**F**) cells transfected with different siRNAs after irradiation. * *P* < 0.05, ** *P* < 0.01, *** *P* < 0.001, and ns *P* > 0.05.

Kaplan-Meier curves showed that both FLOT1 and BHMT could mediate the worse prognosis of glioma patients (Figure 5A-B). More interestingly, the Gliovis database showed that FLOT1 was positively correlated with SDC1 expression (Figure 5C) and BHMT was positively correlated with TGM2 expression (Figure 5D) in glioma tissues. Spontaneously, we wonder to know the potential interaction between FLOT1 and SDC1, as well as BHMT and TGM2 in the regulation of autophagic flux. Firstly, with immunofluorescence assay, we found that the colocalization of FLOT1 with LAMP2 (lysosomal marker) (Figure 5E, G) and BHMT with LC3 (autophagosome marker) (Figure 5F, H) did occur in the irradiated GBM cells. Furthermore, Western blot assay (Figure 5I-J), mRFP-GFP-LC3 assay (Figure 5K-L) and immunofluorescence assay showed that FLOT1 and BHMT could also enhance the autophagy level in the irradiated GBM cells by increasing the fusion process of autophagosomes and lysosomes (Figure S4A). Meanwhile, knockdown of FLOT1 and BHMT (Figure S4B-C) decreased the survival fraction of irradiated GBM cells (Figure 5M-N). Thus, FLOT1 and BHMT did play a role similar to SDC1 and TGM2 in regulating the autophagy of irradiated GBM cells.

### Bindings of SDC1, TGM2, BHMT and FLOT1 promoted the fusion of lysosomes and autophagosomes in irradiated GBM cells

As target proteins have been confirmed to be involved in the fusion process of autophagosome and lysosomes, we selected four time points (1, 2, 4, and 8 h after irradiation) to investigate when GBM cells underwent this fusion significantly. The mRFP-GFP-LC3 assay showed that there were minimal autolysosomes (red dots in overlays) at 1 h and 2 h, but a large amount of them occurred at 4 h and remained constant at 8 h after irradiation (Figure S5A-B). Therefore, we concluded that these four proteins have functioned to ensure the fusion of autophagosome and lysosome at 4 h after irradiation, guaranteeing the integrity process of autophagic flow. To further clarify the interaction between these four proteins and how they are involved in the dynamic process of autophagy flow, we explored the locations of SDC1, TGM2, FLOT1 and BHMT in GBM cells at different time points after irradiation. Considering that our previous studies have found that irradiation can promote the migration of SDC1 carrying TGM2 from the cytomembrane to the cytoplasm through internalization 20, we no longer focus on protein distribution in the unirradiated state, but directly focus on the subsequent changes after irradiation in this study.

**Figure 6:**
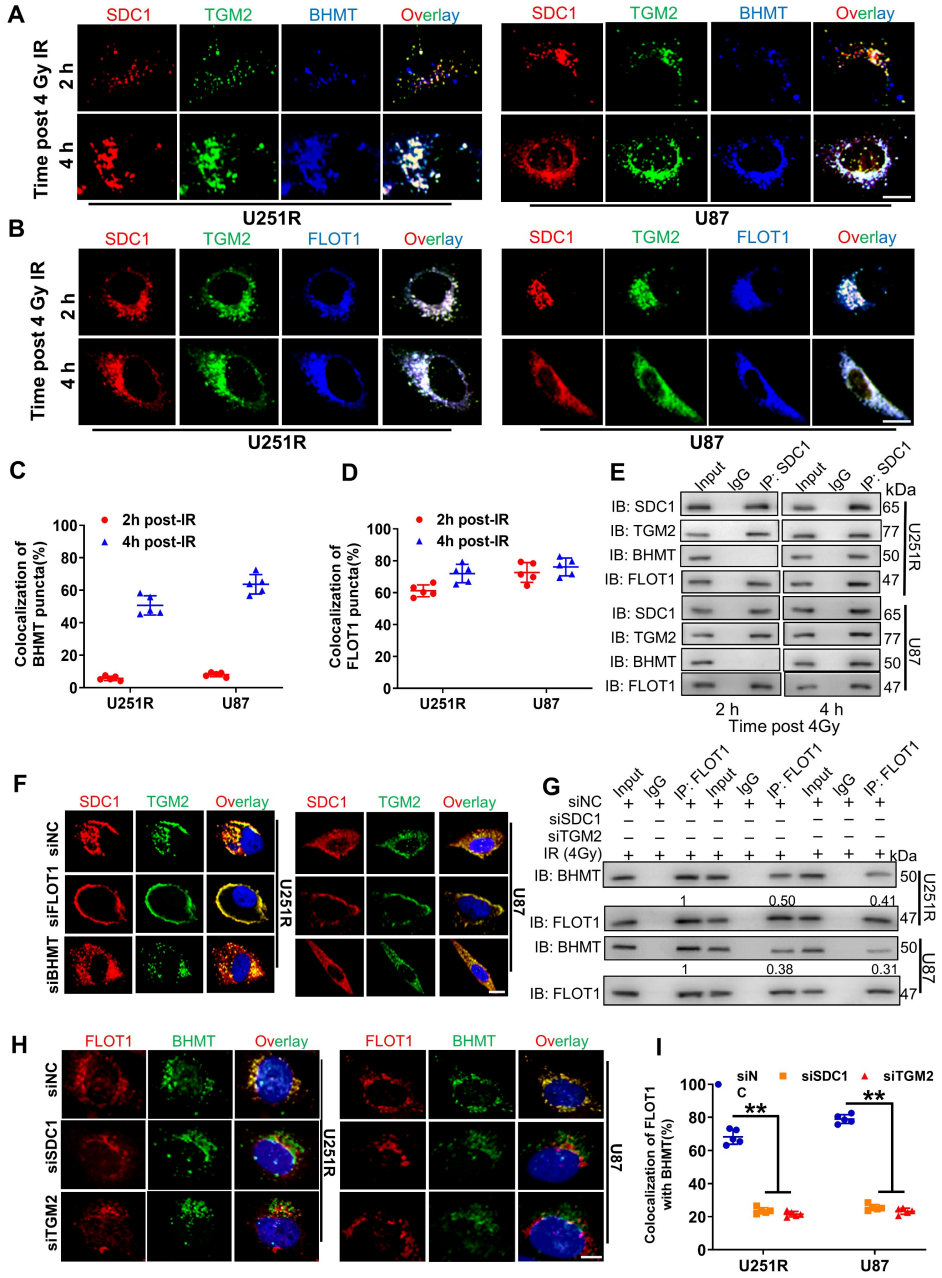
** Bindings of SDC1, TGM2, BHMT and FLOT1 promoted the fusion of lysosomes and autophagosomes in irradiated GBM cells.** (**A-B**) Immunofluorescence images of subcellular location of SDC1 (red), TGM2 (green) and BHMT (blue) (**A**) or FLOT1 (blue) (**B**) proteins in U251R and U87 cells at 2 h and 4 h after 4 Gy irradiation. Scale bars: 10 μm. (**C-D**) Colocalizations of SDC1-TGM2-BHMT (**C**) and SDC1-TGM2-FLOT1 (**D**) were quantified. A total of 50 cells from random fields of each group were counted for each analysis. (**E**) Co-IP assay of TGM2, BHMT and FLOT1 bond with SDC1 in the U251R and U87 cells at 2 h and 4 h after 4 Gy irradiation. (**F**) Immunofluorescence images of subcellular location of SDC1 (red) and TGM2 (green) proteins in U251R and U87 cells transfected with siNC, siFLOT1 or siBHMT at 4 h after 4 Gy irradiation. DAPI-stained nuclei are blue. Scale bars: 10 μm. (**G**) Co-IP assay of BHMT bond with FLOT1 in 251R and U87 cells transfected with siNC, siSDC1 or siTGM2 at 4 h after 4 Gy irradiation. (**H-I**) Immunofluorescence images of subcellular location of FLOT1 (red) and BHMT (green) proteins in U251R and U87 cells transfected with siNC, siSDC1 or siTGM2 at 4 h after 4 Gy irradiation. Scale bars: 10 μm (**H**). Colocalization of FLOT1 and BHMT was quantified (**I**). A total of 50 cells from random fields of each group were counted for each analysis. ** *P* < 0.01.

The immunofluorescence staining showed that, at 1-2 h after 4 Gy irradiation, TGM2 was colocalized with SDC1, but BHMT did not co-localize with SDC1-TGM2 until 4 h after irradiation (Figure 6A, C and S5C, E). Unlike BHMT, FLOT1 has been already colocalized with SDC1-TGM2 since 1-2 h after irradiation (Figure 6B, D and S5D, F). At 8 h after irradiation, both FLOT1 and BHMT maintained co-localization with SDC1-TGM2 (Figure S5C-F). Furthermore, co-IP assay showed that SDC1-TGM2 complex firstly bond to FLOT1 (2 h after irradiation) and then to BHMT (4 h after irradiation) to form a tetramer (Figure 6E). Moreover, we observed that knockdown of FLOT1 blocked the transfer of SDC1-TGM2 from the membrane to the cytoplasm, while knockdown of BHMT had no effect on this process (Figure 6F). Meanwhile, knocking down either SDC1 or TGM2 reduced the co-localization of FLOT1 and BHMT (Figure 6G-I). Conclusively, these results suggested that SDC1 carrying TGM2 firstly bond with FLOT1 on lysosomes after irradiation, and then TGM2 recognized BHMT on autophagosomes to form SDC1-TGM2-FLOT1-BHMT complex, then promoting the encounter of lysosomes and autophagosomes.

### FLOT1 bond to the second constant range in cytoplasmic domain of SDC1, allowing the translocation of SDC1-TGM2 complex to lysosome after irradiation

**Figure 7:**
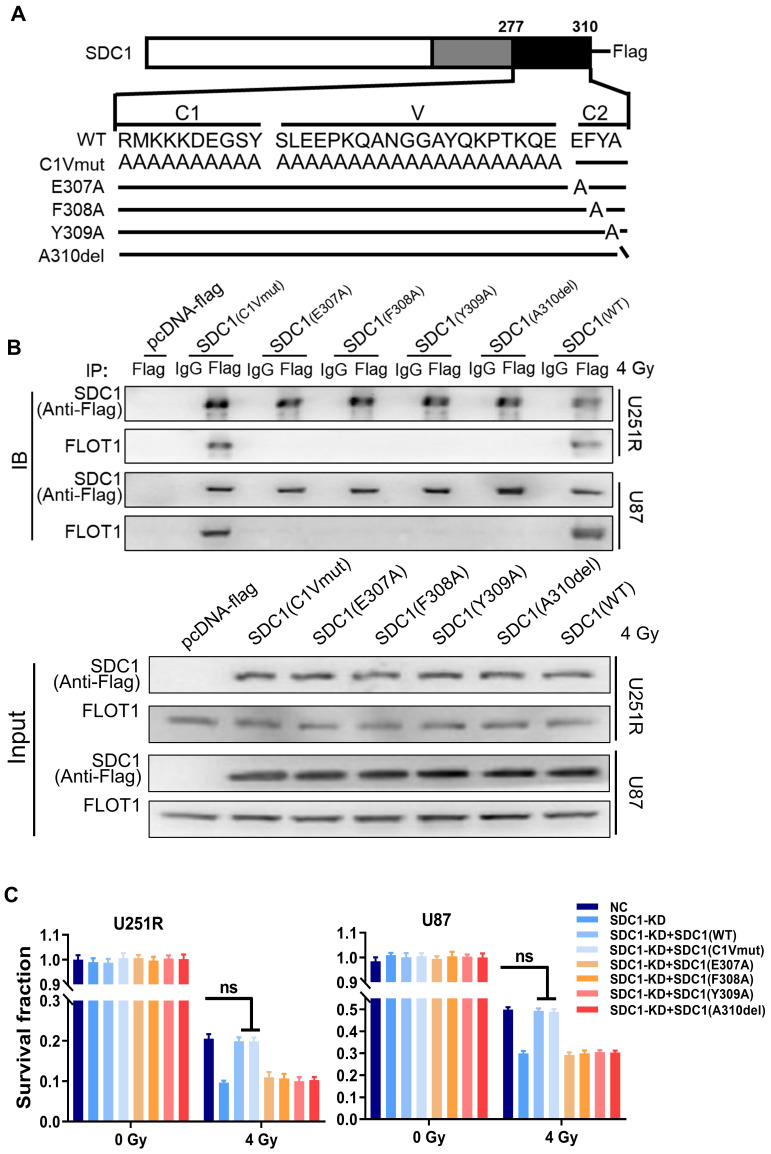
**FLOT1 bond to the second constant range in cytoplasmic domain of SDC1 to regulate radioresistance of GBM cells.** (**A**) Schematic diagram depicting six flag-fused SDC1 proteins (wild-type and mutants). (**B**) Co-IP assay was used to validate the binding of FLOT1 to wild-type and mutant SDC1. (**C**) Survival factions of U251R and U87 cells with SDC1 knocked-down and transfected with different SDC1 mutant plasmids under 0 Gy or 4 Gy irradiation. ns *P* > 0.05.

It has been reported that SDC1 protein has three domain organizations: a single ectodomain, transmembrane and cytoplasmic domains, where the cytoplasmic domains can be subdivided into two constant regions and one variable region 21. To know which region of SDC1 is responsible for the interaction of SDC1-TGM2 complex with FLOT1, we designed six constructs in pcDNA3(-) expression plasmid, where one construct encoded wild-type (WT) human SDC1, one encoded domain-mutant fragment (C1Vmut), and the other four encoded site-directed mutants (E307A, E308A, Y309A and A310del) of human SDC1 (Figure 7A).

Co-IP assay disclosed that the SDC1 fragments containing the second constant range (SDC1-WT and SDC1-C1Vmut) but not the variable region nor the first constant range (SDC1-E307A, SDC1-E308A, SDC1-Y309 and SDC1-A310del) of cytoplasmic domain could bind to FLOT1 in GBM cells (Figure 7B). Western blot analysis and mRFP-GFP-LC3 assay showed that only when the second constant range in the cytoplasmic domain of SDC1 was intact, the irradiated U251R and U87 cells could maintain a high level of autophagic flux (Figure S6A-C). In addition, the rescue experiments also indicated that when SDC1 was knocked down, only the cells transfected with plasmid containing the second variable region of cytoplasmic domain could restore their survival fractions after irradiation (Figure 7C). These findings confirmed that it was the second constant range of cytoplasmic domain of SDC1 that combined with FLOT1 to allow the translocation of SDC1-TGM2 complex to lysosome, then participating in the fusion process of autophagosomes and lysosomes to regulate the radioresistance of GBM cells.

## Discussion

Glioblastoma is an intractable malignant tumor with poor response to radiotherapy and the molecular mechanism of GBM radioresistance is still unsettled. In this study, we revealed that SDC1 and TGM2 played a radioresistance-promoting role in GBM by assisting the fusion of autophagosomes and lysosomes, and upregulating the level of autophagy. We also identified other two molecules, FLOT1 and BHMT, as auxiliary factors that participated in this process of GBM cells, and elucidated their complex molecular mechanisms in conjunction with SDC1 and TGM2 to co-regulate autophagy. Autophagy is a conserved cellular homeostasis mechanism, utilized to maintain homeostasis and manage lipid metabolism 22. Activated by various stresses, autophagic membrane structures are formed to engulf and degrade intracellular structures including damaged organelles, unfolded proteins, and pathogens 23. It has long been understood that autophagy acts as a double-edged sword in regulating cancer cell survival by preventing tumor occurrence but promoting tumor progression and therapy resistance 24, connected to tumor growth, epithelial-mesenchymal transition, metabolism, immunotherapy and chemoresistance mechanisms in a variety of models.

Luo et al. found that long noncoding RNA (lncRNA) EIF3J-DT induces chemoresistance of gastric cancer via autophagy activation 25. Yamamoto et al. indicated that autophagy promotes immune evasion of pancreatic cancer by degrading MHC-I 26. Yang et al. reported that BTN3A1 promotes tumor progression and radiation resistance in esophageal squamous cell carcinoma by regulating ULK1-mediated autophagy 27. Wang et al. also found that PLK1 inhibition enhances the radiosensitivity of breast cancer cells in a manner associated with the suppression of radiation-induced autophagy 28. Our previous research also provided evidence that autophagy was involved in the radioresistance of tumor cells 16, 17. It is evident that autophagy could maintain the survival of tumor cells after radiation and eventually led to radioresistance and malignancy recurrence 29-31. Consistent with these findings, this study also confirmed that autophagy can make GBM more resistant to radiotherapy.

The typical process of autophagy includes the formation of autophagosomes, autophagosome-lysosome fusion, and lysosomal degradation, occurring in a stepwise manner and sequential membrane remodeling involved. According to the current investigation, the different stages of autophagy participates in mediating radioresistance in tumors. For example, Huang et al. reported that ATG4B stimulated autophagy by promoting autophagosome formation through reversible modification of ATG8, resulting tumorigenicity and radioresistance in glioblastoma 32. Meanwhile, hypoxia-induced acetylation of PAK1 enhanced autophagy and promoted GBM development via phosphorylating ATG5, a key protein in the fusion process of autophagosomes and lysosomes 33. A review has detailed the concerted actions of multiple regulators of membrane dynamics in autophagosome-lysosome fusion process, mainly including SNAREs, tethering proteins and RAB GTPases 34. Our results demonstrated for the first time that SDC1, TGM2, FLOT1 and BHMT proteins also facilitate autophagosomal-lysosomal fusion and could increase the autophagy level in the irradiated GBM cells.

SDC1, a type I single transmembrane proteoglycans, can be dysregulated in many types of tumors including multiple myeloma, breast cancers, prostate carcinomas, colon cancer, and pancreatic cancer 10. It participates in a variety of pathological processes, mainly including cancer cell proliferation and invasion, angiogenesis, matrix remodeling, and host defense mechanisms 35. But recently, increasing studies have reported that SDC1 could regulate autophagy-related bioprocesses, such as cell membrane and cytoskeleton rearrangements, vesicle trafficking, endocytosis, and macropinocytosis in malignant tumors 10, 36. TGM2 belongs to the family of tissue transglutaminases and it is significantly increased in several types of cancers, including breast, ovarian, prostate, and pancreatic cancers, and thus positively correlated with poor prognosis 37, consistent with our analysis of the public clinical database analysis (Figure 1J). In various cancer entities, the implication of TGM2 in cancer development, progression, metastasis, and cancer stem cell maintenance has long been discussed 38, 39. Moreover, TGM2 could be a factor promoting autophagy 40, strongly upregulated under various stress conditions including tissue injury, inflammation, protein misfolding, oxidative stress, radiation and chemotherapy 11.

Screened out through the TMT analysis and DEGs analysis using GEO database, SDC1 and TGM2 genes were further verified to be remarkably expressed in radioresistant GBM cells (Figure 1-2), and to lead a higher autophagy level under radiation, compared to radiosensitive ones (Figure 3). This suggested that the resistance to irradiation might be dependent on the increased level of autophagy after irradiation, thus rescuing radiation-induced cell death. Our previous works also indicated that the expression of specific proteins was significantly elevated in radioresistant cell lines than their radiosensitive parent cell line 17, 41-43, which is similar to other findings 44, 45. From subsequent experiments, we learned that the fusion of autophagosomes with lysosomes was markedly shut down once SDC1 or TGM2 was silenced, and the radioresistance was accordingly decreased (Figure 4). Meanwhile, we identified two “partners” of SDC1 and TGM2 in regulating autophagic activity. Some studies found that FLOT1, a lipid raft-associated scaffolding protein acting as an endosomal transporter of membrane protein physiologically, could localize to endo-lysosomes 18. BHMT was considered as a potential cargo-based end-point marker for mammalian autophagy, and could influence the maturation of autophagosomes 15. Our experiments revealed how these four protein molecules of SDC1, TGM2, FLOT1 and BHMT interacted with each other. Through immunofluorescence and co-IP assays, we found that the first step of this interaction was the binding of SDC1, TGM2 and FLOT1 in GBM cells within 2 h after irradiation. Afterwards, BHMT joined in this protein complex to form a copolymer of SDC1-TGM2-FLOT1-BHMT, which ultimately determined the fusion of autophagosome with lysosome.

**Figure 8:**
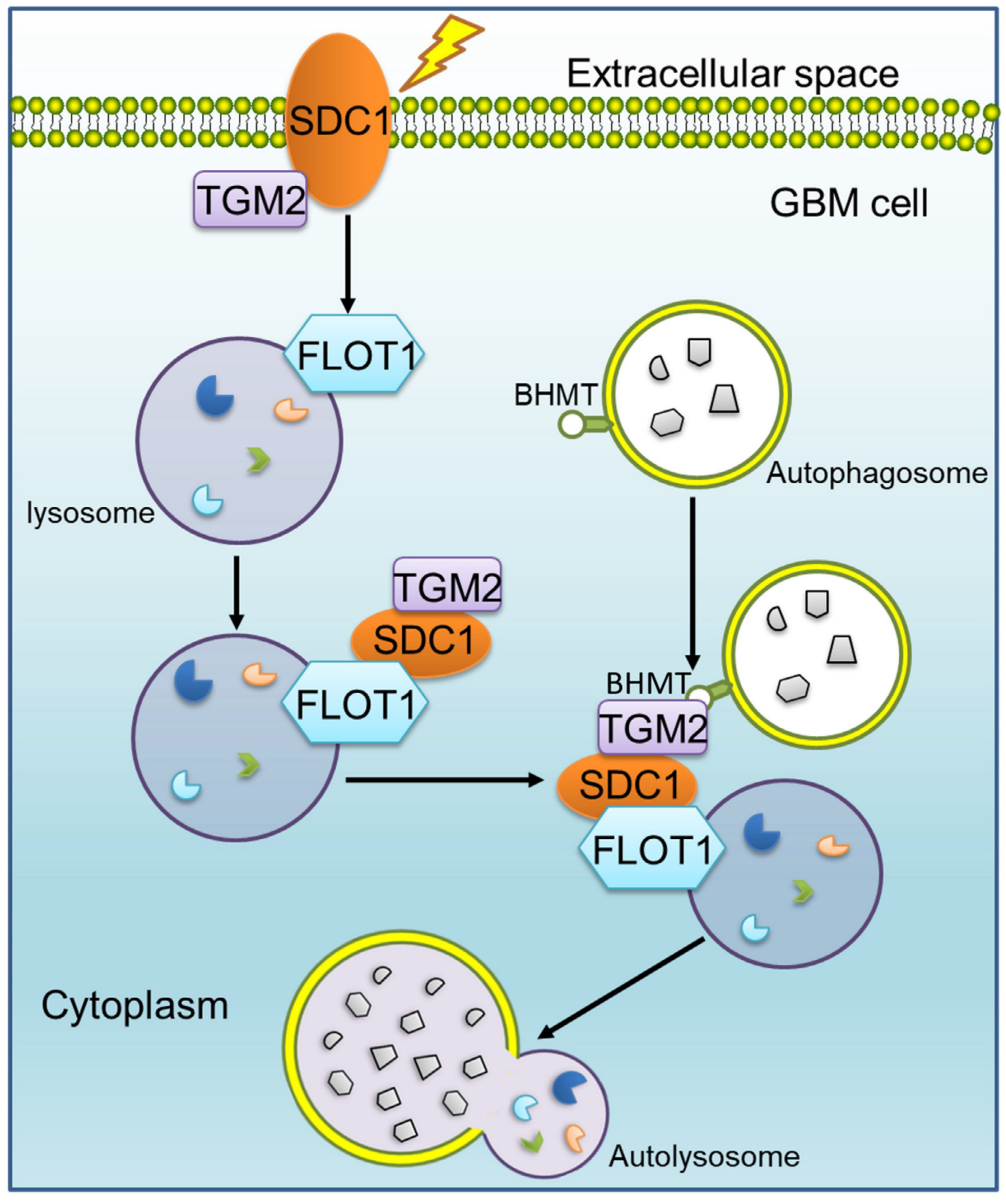
** Mechanism diagram shows how SDC1, TGM2, FLOT1 and BHMT mediate autophagosome-lysosome fusion in GBM cells after irradiation.** In radioresistant cells, SDC1 carried TGM2 from cell membrane into cytoplasm and bonded with FLOT1 located on lysosomal membrane. TGM2 then bonded to BHMT located on autophagosomal membrane, promoting the fusion of autophagosome and lysosome. The increase of autolysosome ultimately resulted in the enhancement of autophagy level and radioresistance.

Our previous study has reported that SDC1 on the surface of GBM cells could carry TGM2 from cell membrane to lysosome, but it is not clear which molecule on lysosome directly combines with SDC1 20. Given that FLOT1 is located on lysosome, we found for the first time that there was a binding relationship among SDC1, TGM2 and FLOT1 at 1-2 h after irradiation. Besides, knockdown of FLOT1 blocked the membrane translocation of SDC1-TGM2 (Figure 6F). Thus, FLOT1 was the molecule that directly bound to SDC1 in the SDC1-TGM2-FLOT1 complex. Furthermore, we confirmed that it was the second constant range of cytoplasmic domain of SDC1 that combined with FLOT1 by constructing plasmids expressing different SDC1 domains (Figure 7). Back to the autophagy flow process, 4 h after irradiation, the SDC1-TGM2-FLOT1 complex could bind to BHMT on the autophagosomes to form a tetramer, which further prompts the encounter between lysosomes and autophagosomes. But curiously, which molecule is directly combined with BHMT, SDC1, TGM2 or FLOT1? From our analysis above, the role of TGM2 in tetramers has not been discussed yet, but the aforementioned results have confirmed that just knockdown of TGM2 could also restrain the fusion of lysosomes and autophagosomes (Figure 4). Through co-IP experiments, we found that FLOT1 could not bind to BHMT when TGM2 was absent (Figure 6G-I), indicating that TGM2 acted as a bridge for binding BHMT to the complex formed in the previous step. Moreover, it has been reported that TGM2 can directly recognize BHMT on the autophagosomal membrane 46. Therefore, TGM2 was the most potential molecule binding to BHMT directly. SDC1 is the key initiator in the whole formation process of the copolymer of SDC1-TGM2-FLOT1-BHMT. If SDC1 was missing, radiation could not recruit together of TGM2 and FLOT1, not to mention BHMT, to draw lysosomes to encounter autophagosomes (Figure 8).

Ultimately, our report revealed that the SDC1-TGM2-FLOT1-BHMT copolymer played a significant role in promoting radioresistance of GBM by assisting the fusion of autophagosomes and lysosomes. A recent study reported that regorafenib can inhibit RAB11A-mediated autophagosome-lysosome fusion, resulting in lethal autophagy arrest in GBM cells 47. In those study, the target molecules, participating in autophagy regulation, share a similar process with ours, both of which enhance autophagy flux by promoting the fusion of autophagosomes and lysosomes, however, the final therapeutic effects on tumors are completely opposite. Jiang et al. suggested that an increase in autophagic flux contributed to eliminate tumors, whereas we declared that the elevated level of autophagy promoted tumor progression and therapy resistance. Possibly, chemotherapy drugs and X-ray irradiation may induce autophagy with different mechanisms in GBM, suggesting that although our current study observed the SDC1-TGM2-FLOT1-BHMT copolymer mediating radioresistance in GBM cells by promoting autophagic flux, it remains to be further explored whether this tetramer plays a similar role in the process of autophagy induced by other stimuli in other types of tumors.

## Conclusions

In summary, we identified a new protein copolymer SDC1-TGM2-FLOT1-BHMT involved in the fusion of lysosomes and autophagosomes and disclosed that it contributed to the maintenance of autophagic flux in the irradiated tumor cells and ultimately enhanced the radioresistance of GBM. SDC1-TGM2-FLOT1-BHMT may represent a novel potential sensitization target for GBM radiotherapy, which could be further confirmed by additional in vivo experiments and clinical trials in future.

## Supplementary Material

Supplementary figures and tables.

## Abbreviations

GBM: Glioblastoma
TMT: Tandem mass tag
SDC1: Syndecan 1
TGM2: Transglutaminase 2
AMPK: Adenosine monophosphate-activated protein kinase
mTOR: mammalian target of rapamycin
TEM: Transmission electron microscopy
Baf-A1: bafilomycin A1
3-MA: 3-Methyladenine
FLOT1: Flotillin 1
BHMT: Betaine Homocysteine Methyltransferase
CNS: central nervous system
GEO: Gene Expression Omnibus
DEGs: differentially expressed genes
