# Supplementary material for: SDC1-TGM2-FLOT1-BHMT complex determines radiosensitivity of glioblastoma by influencing the fusion of autophagosomes with lysosomes
Source: Theranostics. 2023 Jun 26;13(11):3725–43. doi: 10.7150/thno.81999 (PMC10334832; doi:10.7150/thno.81999)
Supplement: Supplementary file 1 — Supplementary figures and tables. [file thnov13p3725s1.pdf]

Supplementary Figures and Tables

A

Gene Name	U251(IR/nonIR) (A)	U87(IR/nonIR) (B)	U251R/U251 (C)	Tumoral/Normal (D)	Significance A	Significance B	Significance C	Significance D
STAT1	1.669018562	3.679977228	2.048877147	4.58959735	0.0192	0.00162	1.27E-10	8.48E-08
BST2	1.747145792	4.575417504	1.880758808	2.31998182	0.0155	0.0215	1.61E-08	0.0000998
ERAP1	2.602683711	3.600120373	1.534666667	2.204677525	0.0000582	0.00145	0.00014786	0.00000228
SOD2	2.070529848	3.129735398	1.421052632	2.21048176	0.00149	0.003	0.00198041	0.00000486
SDC1	1.731473131	3.661778422	1.340341656	1.584458617	0.0122	0.0293	0.0104712	0.0000798
TGM2	2.789487333	3.980861077	1.313343328	2.29234413	0.0000365	4.81E-03	0.017583	0.00000097

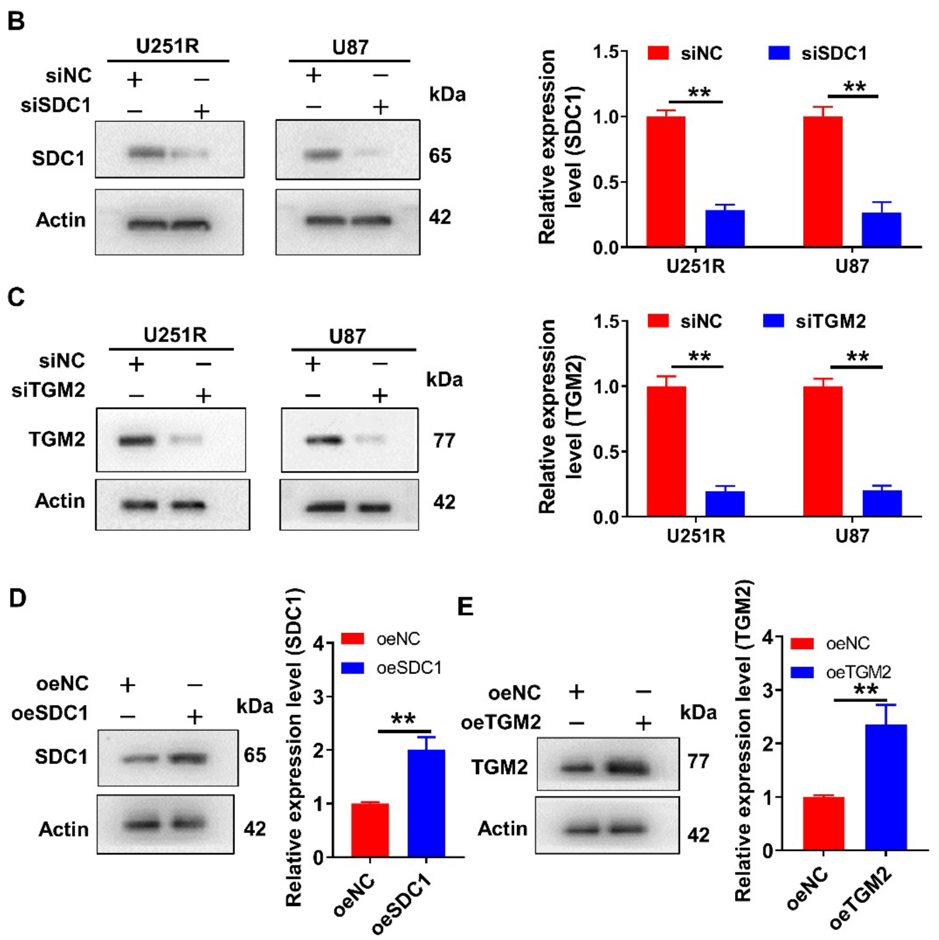

Figure S1. Efficiency of siSDC1/siTGM2 and oeSDC1/oeTGM2 transfection in GBM cells. (**A**) List of the 6 upregulated genes from Figure 1G. (**B-C**) Western blot analysis (left) and quantitation (right) of SDC1 (**B**) and TGM2 (**C**) expression levels in U251R and U87 cells transfected with siNC, siSDC1 or siTGM2. (**D-E**) Western blot analysis (left) and quantitation (right) of SDC1 (**D**) and TGM2 (**E**) expression levels in U251 cells transfected with plasmid-mediated SDC1 or TGM2 overexpression (oeNC, oeSDC1 or oeTGM2). Actin was used as a loading control. ** $P < 0.01$.

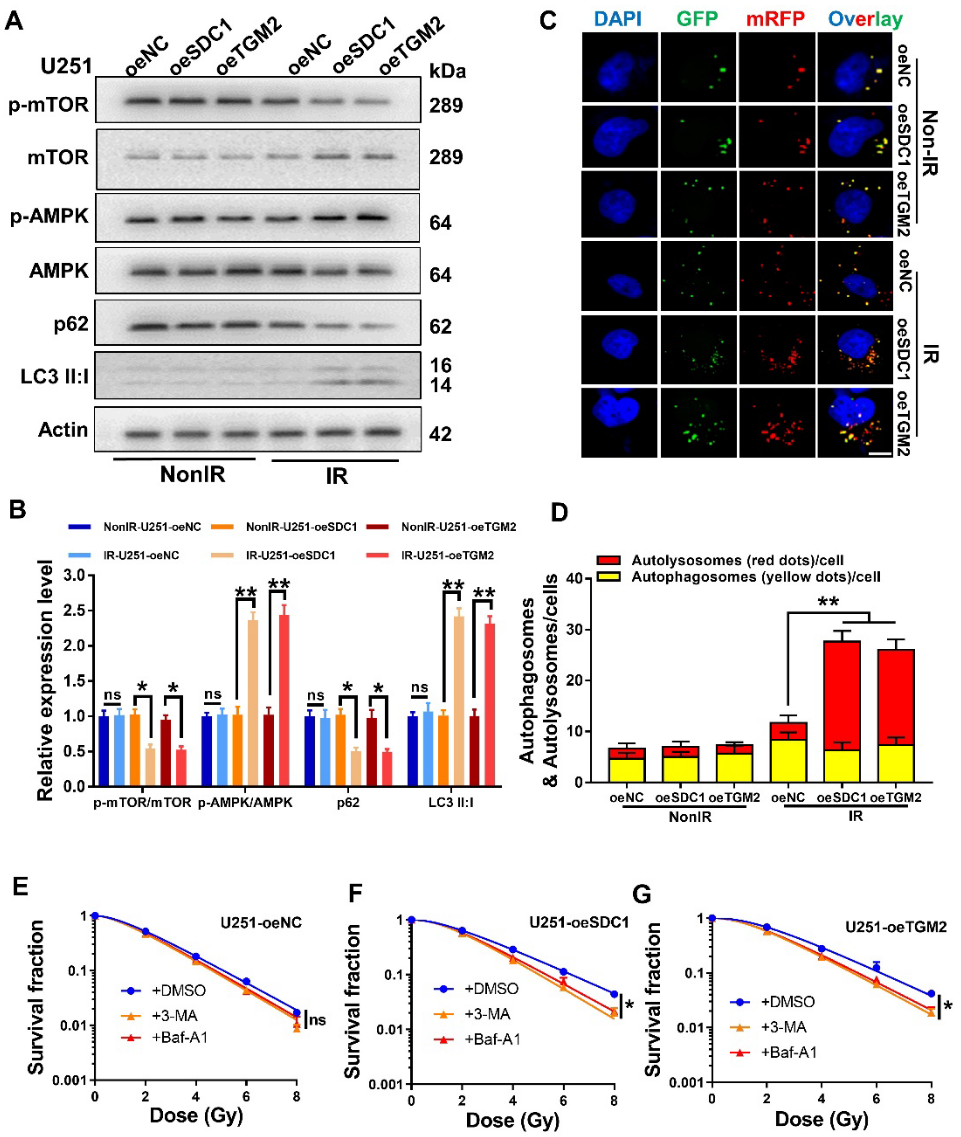

Figure S2. Overexpression of SDC1 and TGM2 enhanced the radioresistance of GBM cells by upregulating the level of autophagy. U251 cells transfected with plasmid-mediated SDC1 or TGM2 overexpression (U251-oeNC, U251-oeSDC1 or U251-oeTGM2). (**A-B**) Western blot analysis (**A**) and quantitation (**B**) of p-mTOR/mTOR, p-AMPK/AMPK, p62 and LC3-II:I protein levels in U251-oeNC, U251-oeSDC1 and U251-oeTGM2 cells at 4 h after 4 Gy irradiation. (**C-D**) Fluorescence images of U251-oeNC, U251-oeSDC1 and U251-oeTGM2 cells transfected with mRFP-GFP-LC3-tagged adenovirus (×40) after 4 Gy irradiation. Red dots indicate autolysosomes while yellow dots indicate autophagosomes in overlays. Nuclei were stained with DAPI. Scale bars: 10 μm. The average number of autophagosomes and autolysosomes in each indicated cell was quantified. A total of 50 cells from random fields of each group were counted for each analysis. (**E-G**) Clonogenic survivals of U251-oeNC (**E**), U251-oeSDC1 (**F**), and U251-oeTGM2 (**G**) cells. Cells were pre-treated with 100 nM 3-Methyladenine (3-MA) and bafilomycin A1 (Baf-A1) for 2 h and then irradiated with X-rays. * $P < 0.05$, ** $P < 0.01$, and ns $P > 0.05$.

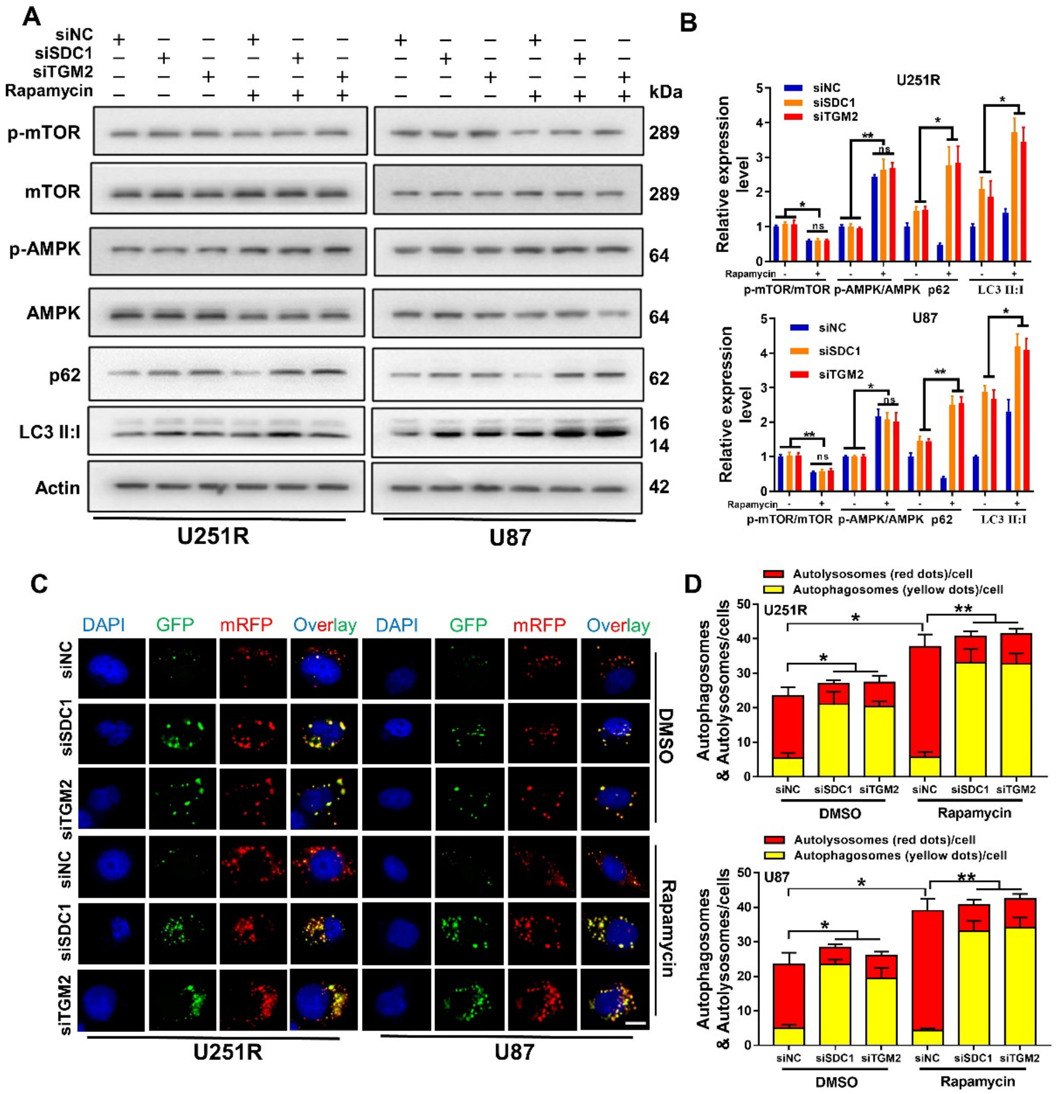

Figure S3. Autophagic flux was truncated by the knockdown of SDC1 and TGM2 in GBM cells. (**A-B**) Western blot analysis (**A**) and quantitation (**B**) of p-mTOR/mTOR, p-AMPK/AMPK, p62 and LC3-II:I protein levels in different siRNAs transfected U251R and U87 cells at 4 h after 4 Gy irradiation. The cells were pre-treated with 50 nM rapamycin for 2 h prior to irradiation. (**C-D**) Fluorescence images of U251R and U87 cells transfected with different siRNAs and mRFP-GFP-LC3-tagged adenovirus (×40), that were pre-treated with 50 nM rapamycin for 2 h and then irradiated with X-rays. Red dots indicate autolysosomes while yellow dots indicate autophagosomes in overlays. Nuclei were stained with DAPI. Scale bars: 10 μm. The average number of autophagosomes and autolysosomes in each indicated cell was quantified. A total of 50 cells from random fields of each group were counted for each analysis. * $P < 0.05$, ** $P < 0.01$, and ns $P > 0.05$.

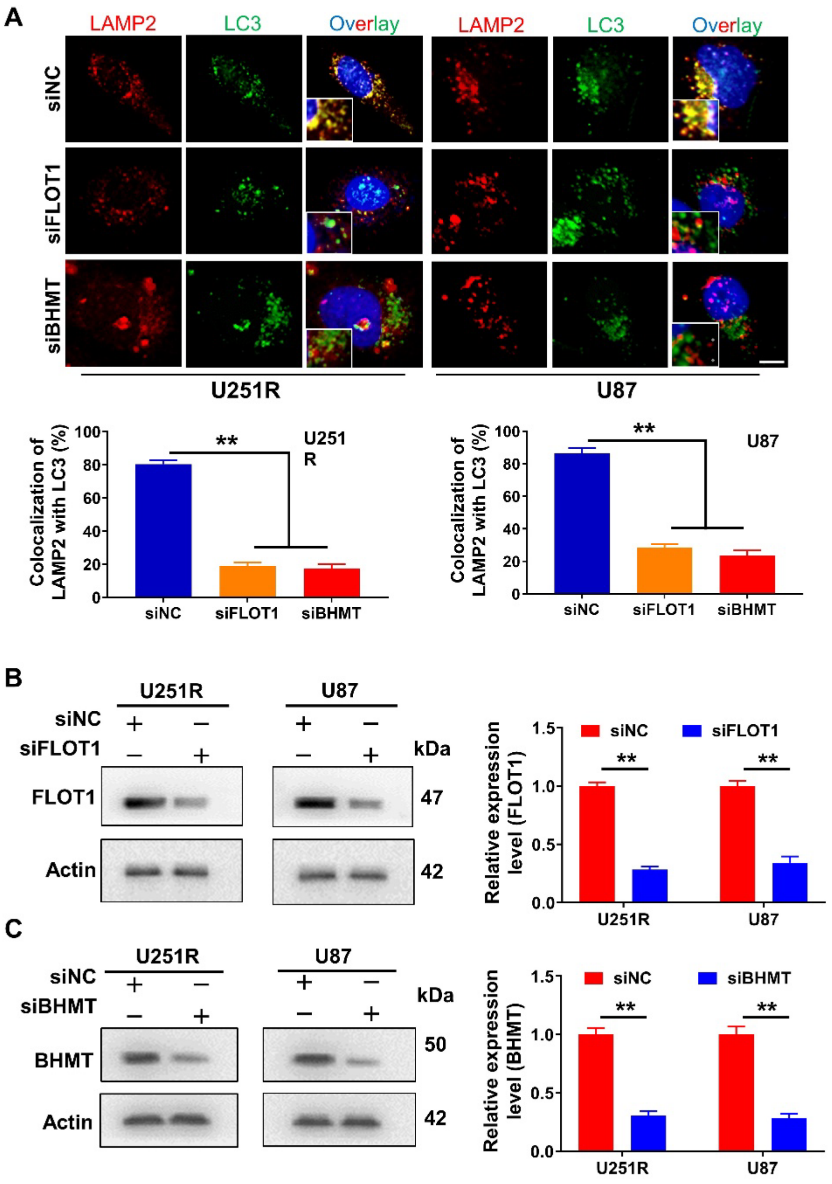

Figure S4. Knockdown of FLOT1 and BHMT reduced autophagosome and lysosome encounter in GBM cells. (**A**) Immunostaining images (up) of LAMP2 (red) and LC3 (green) in different siRNAs transfected U251R and U87 cells at 4 h after 4 Gy irradiation. Nuclei were stained with DAPI (blue). Colocalization of LC3 and LAMP2 was quantified (down). A total of 50 cells from random fields of each group were counted for each analysis. Scale bars: 10 μm. (**B, C**) Western blot analysis (left) and quantitation (right) of FLOT1 (**B**) and BHMT (**C**) expression levels in U251R and U87 cells transfected with siNC, siSDC1 or siBHMT. Actin was used as a loading control. ** $P < 0.01$.

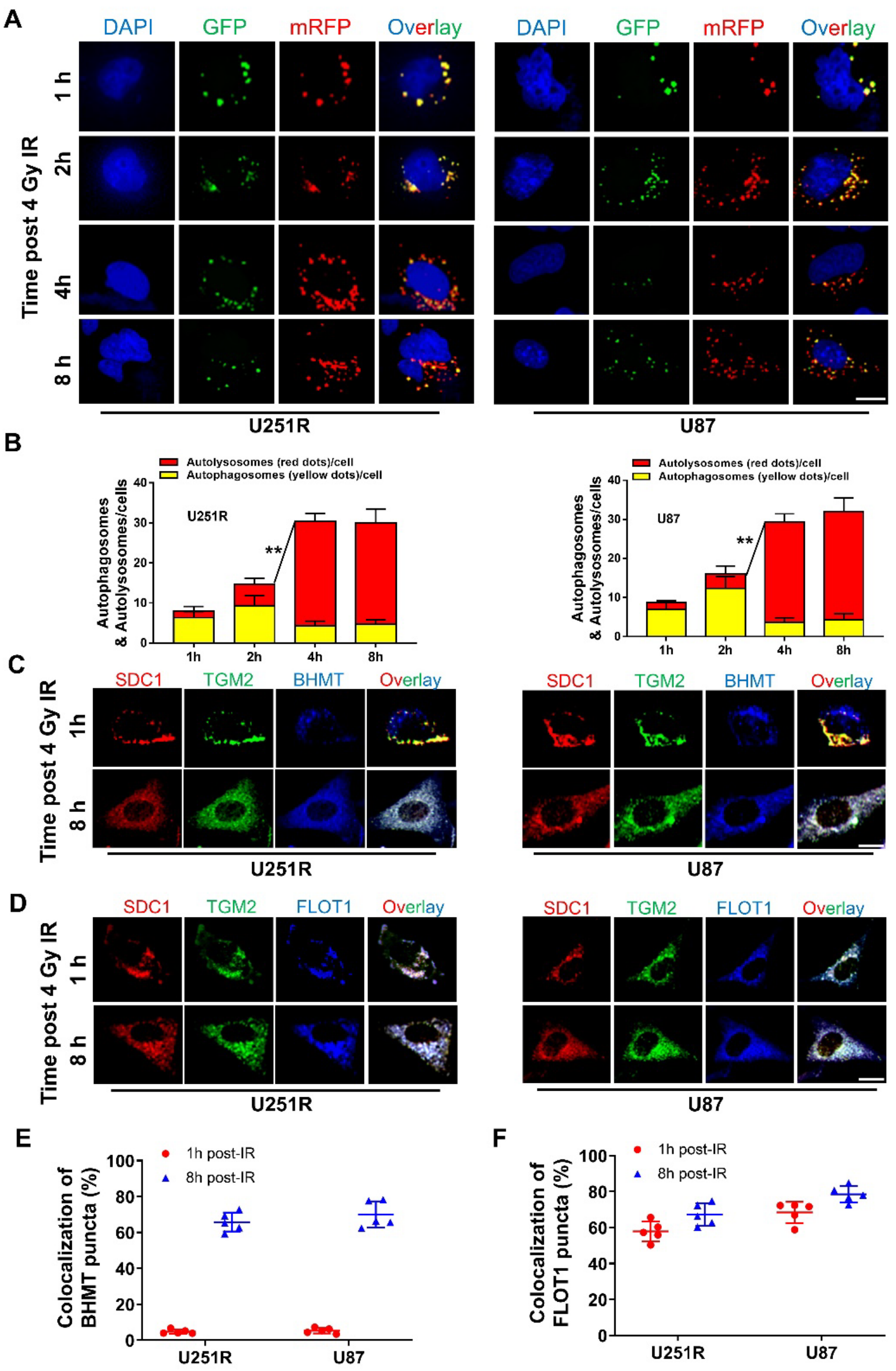

Figure S5. The colocalization of SDC1, TGM2, BHMT and FLOT1 in GBM cells at various time points after irradiation. (**A-B**) Fluorescence images of U251R and U87 cells transfected with different siRNAs and mRFP-GFP-LC3-tagged adenovirus (×40) at 1 h, 2 h, 4 h and 8 h after 4 Gy irradiation. Red dots indicate autolysosomes while yellow dots indicate autophagosomes in overlays. Nuclei were stained with DAPI. Scale bars: 10 μm. The average number of autophagosomes and autolysosomes in each indicated cell was quantified. A total of 50 cells from random fields of each group were counted for each analysis. (**C-D**) Immunofluorescence images of subcellular location of SDC1 (red), TGM2 (green) and BHMT (blue) (**C**) or FLOT1 (blue) (**D**) proteins in U251R and U87 cells at 1 h and 8 h after 4 Gy irradiation. Scale bars: 10 μm. (**E-F**) Colocalizations of SDC1-TGM2-BHMT (**C**) and SDC1-TGM2-FLOT1 (**D**) were quantified. A total of 50 cells from random fields of each group were counted for each analysis. ** $P < 0.01$.

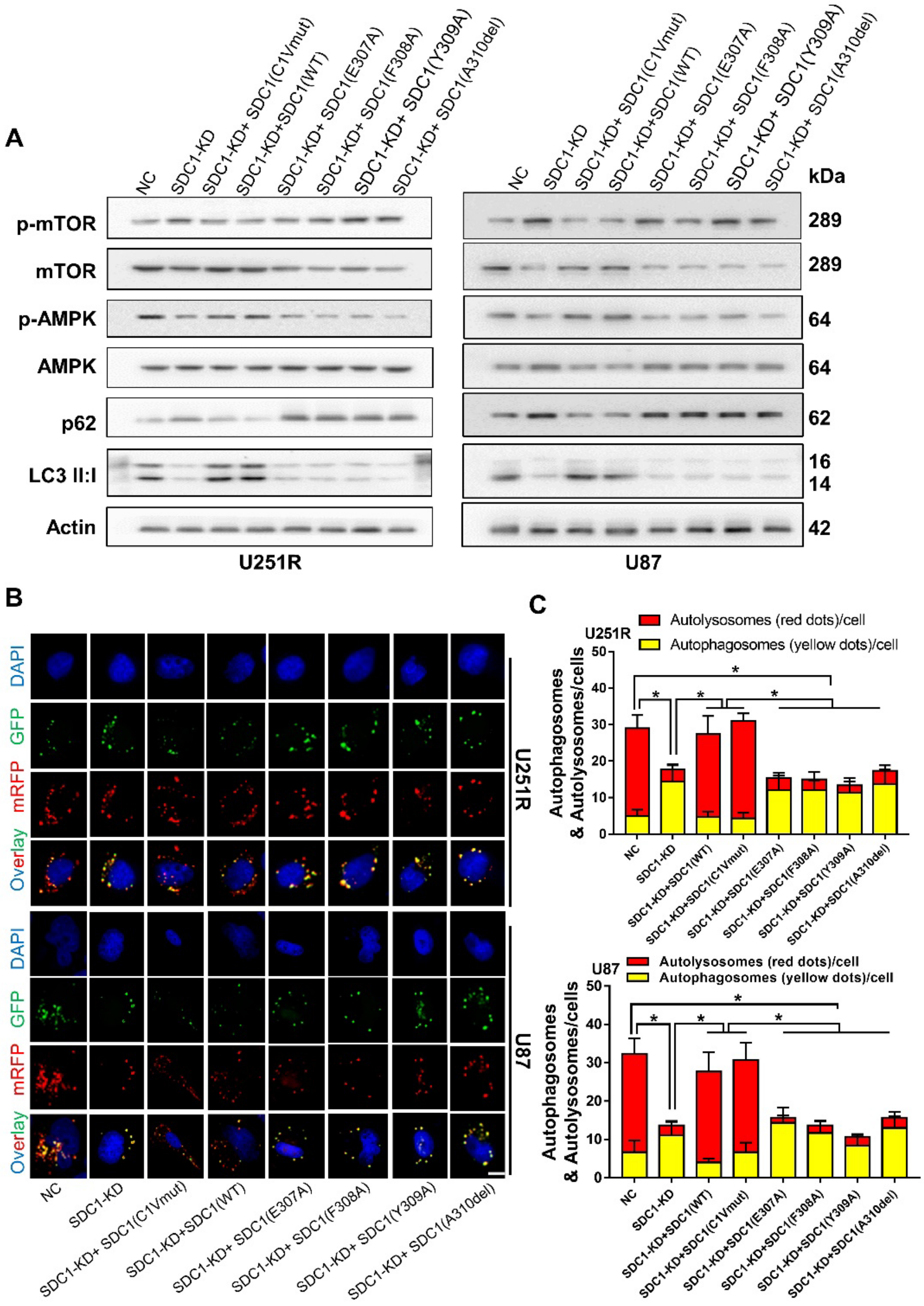

Figure S6. The autophagic flux in U251R and U87 cells expressing SDC1 mutants after irradiation. (**A**) Western blot analysis of p-mTOR/mTOR, p-AMPK/AMPK, p62 and LC3-II:I protein levels in U251R and U87 cells with SDC1 knocked-down and transfected with different SDC1 mutant plasmids at 4 h after 4 Gy irradiation. (**B-C**) Fluorescence images of U251R and U87 cells transfected with SDC1 mutants and mRFP-GFP-LC3-tagged adenovirus (×40) after 4 Gy irradiation. Red dots indicate autolysosomes while yellow dots indicate autophagosomes in overlays. Nuclei were stained with DAPI. Scale bars: 10 μm. The average number of autophagosomes and autolysosomes in each indicated cell was quantified. A total of 50 cells from random fields of each group were counted for each analysis. * $P < 0.05$.

Table S1. Primers used for RT-PCR.

Genes	Forward primer (5'-3')	Reverse primer (5'-3')
STAT1	ATCAGGCTCAGTCGGGGAATA	TGGTCTCGTGTTCTCTGTTCT
BST2	CACACTGTGATGGCCCTAATG	GTCCGCGATTCTCACGCTT
ERAP1	GCAAACCTTACCACGCTGAC	GGTTCTTCCGATAGCCTCTCTC
SOD2	GGAAGCCATCAAACGTGACTT	CCCGTTCCTTATTGAAACCAAGC
SDC1	CCACCATGAGACCTCAACCC	GCCACTACAGCCGTATTCTCC
TGM2	CAAGGCCCGTTTTCCACTAAG	GAGGCGATACAGGCCGATG
β-Actin	CATGTACGTTGCTATCCAGGC	CTCCTTAATGTCACGCACGAT

Table S2. Antibodies used in the experiments.

Antibody	Supplier	Catalog #	Application
SDC1	Proteintech	10593-1-AP	IB (1:1000) IP (1:100)
SDC1	Proteintech	67155-1-Ig	IF (1:200)
TGM2	Proteintech	15100-1-AP	IB (1:1000) IF (1:200)
FLOT1	Proteintech	15571-1-AP	IB (1:1000) IP (1:100) IF (1:200)
BHMT	Proteintech	15965-1-AP	IB (1:1000)
BHMT	Abcam	ab243698	IF (1:200)
p62	Proteintech	18420-1-AP	IB (1:1000)
LC3	Cell Signaling Technology	3868	IB (1:1000) IF (1:200)
LAMP2	Proteintech	66301-1-Ig	IF (1:200)
p-mTOR	ABclonal	AP0115	IB (1:1000)
mTOR	ABclonal	A11355	IB (1:1000)
p-AMPK	ABclonal	AP0883	IB (1:1000)
AMPK	ABclonal	A17290	IB (1:1000)
β-Actin	Proteintech	66009-1-Ig	IB (1:1000)
HRP-anti-mouse	Cell Signaling Technology	7076	IB (1:5000)
HRP-anti-rabbit	Cell Signaling Technology	7074	IB (1:5000)
Alexa Fluor 350-labeled Goat Anti-Mouse IgG	Beyotime	A0412	IF (1:1000)
Alexa Fluor 488-labeled Goat Anti-Rabbit IgG	Beyotime	A0423	IF (1:1000)
Alexa Fluor 555-labeled Donkey Anti-Rabbit IgG	Beyotime	A0453	IF (1:1000)
Alexa Fluor 555-labeled Donkey Anti-Mouse IgG	Beyotime	A0460	IF (1:1000)
Alexa Fluor 647-labeled Goat Anti-Mouse IgG	Beyotime	A0473	IF (1:1000)
Rabbit mAb IgG Isotype control	Cell Signaling Technology	3423	IP (1:100)
Flag	Sigma	F7425	IB (1:1000) IP (1:100)

Table S3. Target sequences of siRNAs.

Genes	Target sense (5'-3')
SDC1	CCGCAAATTGTGGCTACTAAT
TGM2	ACAGCAACCTTCTCATCGAGT
FLOT1	CCAGGACTATTTGCACTCTTT
BHMT	GCAACAGTTAGAGGTCTTTAT
Negative control	TCCTGGAACGTGTTCAACGT
